# The microevolutionary trajectory of endemic multidrug-resistant tuberculosis strains in Portugal toward increased drug resistance levels and its clinical significance

**DOI:** 10.3389/fmicb.2025.1716549

**Published:** 2026-01-21

**Authors:** Pedro Gomes, Paulo Paixão, Fernando Maltez, Laura Brum, Jody E. Phelan, Susana Campino, Taane G. Clark, Miguel Viveiros, Isabel Portugal, João Perdigão

**Affiliations:** 1 Faculdade de Farmácia, Research Institute for Medicines, Universidade de Lisboa, Lisbon, Portugal; 2 Serviço de Doenças Infecciosas, Hospital Curry Cabral, Centro Hospitalar Universitário de Lisboa Central, Lisbon, Portugal; 3 SYNLAB Portugal, Lisbon, Portugal; 4 London School of Hygiene and Tropical Medicine, London, United Kingdom; 5 Global Health and Tropical Medicine, GHTM, LA-REAL, Instituto de Higiene e Medicina Tropical, IHMT, Universidade NOVA de Lisboa, Lisbon, Portugal

**Keywords:** antimicrobial resistance, drug efficacy, endemic transmission, *Mycobacterium tuberculosis*, pharmacokinetics, whole-genome sequencing

## Abstract

Portugal has one of the highest incidence rates of tuberculosis (TB) in Western Europe and, historically, multidrug-resistant (MDR) cases have been strongly associated with *Mycobacterium tuberculosis* strains pertaining to the endemic Q1 and Lisboa3 clades. Notwithstanding, the contribution of drug resistance-associated allelic configurations in these clades to differing levels of drug resistance and their relationship with drug efficacy has yet to be uncovered. A representative sample of the drug-resistant *M. tuberculosis* population in Portugal, comprised of 40 clinical strains were subjected to whole genome sequencing for characterization of allelic combinations of drug resistance-associated mutations and their minimum inhibitory concentrations for 12 anti-TB drugs was determined. Pharmacokinetic (PK) models were generated to ascertain the maximum concentration to which each drug remains efficacious. Drug resistance levels were determined and compared between different allelic configurations. Double *inhA* and *embA/B* mutation genotypes contributed with increased isoniazid and ethambutol resistance levels compared with single mutation configurations, respectively. Significant differences in drug resistance levels were observed between phylogenetic groups for rifamycin, streptomycin and ethionamide, largely explained by the presence/absence of unique high-level resistance-associated genotypes. The PK models for isoniazid and moxifloxacin suggest an increase in dosage to be ineffective against strains harboring high-level resistance-conferring double *inhA* mutations and *gyrA/B* mutations. Cycloserine and para-aminosalicylic acid are the only drugs predicted to remain efficacious against the majority of tested strains, while the effectiveness of newer drugs like bedaquiline, pretomanid and delamanid have yet to be uncovered. Proper diagnosis of drug resistance-associated mutations provides invaluable insights into the treatment of TB, as different allelic configurations lead to differing drug resistance levels, often rendering drugs ineffective.

## 1 Introduction

According to the World Health Organization (WHO), more than 10 million people worldwide developed tuberculosis (TB) in 2024 alone(WHO, 2024). Portugal reported a notification rate of 14.6 cases per 100,000 population in 2022, the highest incidence across Western Europe (ECDC and WHO, 2023).

Multidrug-resistant (MDR) TB, defined as resistance to isoniazid and rifampicin, became a major health concern worldwide since the reported hospital outbreaks during 1988–1991 in the United States (CDC, 1991). Another form of resistance is pre-extensively drug-resistant (pre-XDR) TB, defined as MDR with concomitant resistance to fluoroquinolones and, since 1996, MDR TB and pre-extensively drug-resistant (pre-XDR) TB cases have been reported in Portugal (Perdigão et al., 2020). Historically, in Portugal, the majority of MDR and pre-XDR TB cases have been associated with strains from the predominant Lisboa3 and Q1 endemic clades. Both clades were initially described in the Lisbon Health Region in 1998 and subsequently characterized as SIT20/LAM1/L4.3.4.2 and SIT1106/LAM4/L4.3.4.2, respectively, with earlier studies denoting a varying prevalence across MDR TB clinical isolates ranging between 55.8–71.4% for Lisboa3 and 25.9–28.6% for Q1 strains (Portugal et al., 1999). The importance of understanding the local epidemiology and of the contribution of both clades to primary MDR TB in the country prompted for the implementation of strategies that successfully led to a steady decline in the MDR TB incidence in Portugal, with 13 cases notified in 2023, of which 3 were pre-XDR TB. Nonetheless, and according to WHO data, the treatment success rate for MDR TB in Portugal in 2022 was 55%, below the 68% average rate for the WHO European Region and the global success rate estimated at 71% (ECDC and WHO, 2023).

The characterization of drug resistance-associated mutations in clinical isolates from Portugal has since continued, with many drug-resistant strains harboring unique combinations of mutations, some only found in strains from specific genetic clusters (Perdigão et al., 2010; Perdigão et al., 2014). A small number of mutations have been correlated with drug resistance levels. Notably, there were the vastly differing contributions of prevalent *rrs* and *eis* mutations toward increased resistance levels for the second-line injectable agents amikacin (AMK) and kanamycin (KAN)(Perdigão et al., 2013), and the contribution of specific sets of *inhA* double mutations toward high-level isoniazid (INH) and ethionamide (ETO) resistance (Machado et al., 2013). The diversity of drug resistance-associated mutations, for which drug resistance levels is known is very limited for Portuguese strains, particularly across the microevolutionary trajectory of the endemic clades, where only a few drugs having been tested.

Cotemporary whole-genome sequencing (WGS) technologies have proven indispensable in the study of *M. tuberculosis* biology and epidemiology (Niemann et al., 2009). Particularly, the capacity to discriminate at the single nucleotide polymorphism (SNP) level has enabled the directional reconstruction of transmission events while simultaneously allowing for the reconstruction of the microevolutionary events within the host, between transmission or those leading the successful accumulation of resistance associated variants (do Carmo Guimarães et al., 2021; Merker et al., 2013; Cohen et al., 2015; Walker et al., 2013). Together with the complete record of the reference genome of the H37Rv strain, full-spectrum diagnosis of drug resistance-associated mutations is now a possibility (Cole et al., 1998). WGS was effectively used in deciphering the evolutionary events that led to drug resistance acquisition in MDR and pre-XDR clinical strains from Portugal, and contributed to the discovery of unique sets of mutations found predominantly in each of the two previously mentioned genetic clades (Perdigão et al., 2014). This study aims to characterize drug resistance-associated mutations of clinical isolates from Portugal at the genome level, uncover the effects of differing allelic configurations with additive levels of drug resistance, and correlate with drug efficacy through the implementation of pharmacokinetic (PK) models.

## 2 Methods

### 2.1 Clinical isolates and drug susceptibility testing (DST)

A sample of 40 *M. tuberculosis* clinical isolates, each corresponding to a different patient were selected for this study, obtained from hospitals and laboratories across Portugal, between 2008 and 2016. The sample consists of a convenience sample selected purposely to include different allelic configurations previously detected in Portugal and reflect the diversity of endemic clades circulating in the country, with special focus on Lisboa3 and Q1 isolates. As such, among all isolates the sample includes but is not limited to: 8 Lisboa3 isolates, of which 2 are non-MDR, 3 MDR and 3 pre-XDR; 7 Q1 isolates, of which 1 is non-MDR, 4 MDR and 2 pre-XDR; and, 5 SIT1/Beijing isolates, of which 1 is non-MDR, 3 MDR and 1 pan-susceptible. In total the sample also includes 6 pan-susceptible isolates to provide baseline resistance levels and phylogenetic context across the phylogenetic tree.

Drug resistance phenotypes were determined by DST using the Becton Dickinson (BD) BACTEC Mycobacterial Growth Indicator Tube (MGIT) 960 System against 14 first- and second-line anti-TB drugs (Supplementary Table 1) as per the manufacturer’s instructions. Briefly, the mycobacteria were grown in MGIT tubes until the growth unit (GU) reached 100–200 and used as the inoculum. MGIT tubes were prepared with dilutions of each compound according to standardized critical concentrations (Supplementary Table 2). Two drug-free controls were included with each test; one inoculated with 0.5 mL of the suspension (absolute control) and the other inoculated with 0.5 mL of a 1: 100 dilution of the suspension (proportional control; 1: 10 for pyrazinamide). The vials were incubated at 37 °C and monitored in the BACTEC MGIT 960 system equipped with the TB eXIST module, every hour until the GU in the proportional control reached 400 (Viveiros et al., 2002; Springer et al., 2009). Clinical isolates resistant to INH and rifampin (RIF) were categorized as MDR, and MDR isolates resistant to at least one fluoroquinolone were categorized as pre-XDR (WHO, 2021).

### 2.2 WGS, variant calling and classification

Clinical isolates were incubated on Löwenstein-Jensen (LJ) media slants, and genomic DNA was subsequently extracted using the cetyl trimethylammonium bromide protocol (van Soolingen et al., 2001). WGS was performed by Illumina HiSeq 2500/4000 sequencing platforms through the Applied Genomics Centre at the London School of Hygiene and Tropical Medicine, using paired-end mode, producing 100/150 bp reads (BioProject accession PRJEB47098). Quality control of the reads was enacted using the Trimmomatic software (v0.39). Alignment of trimmed reads to the *M. tuberculosis* H37Rv strain reference genome (GenBank accession NC000962.3) was performed using the Burrows-Wheeler Alignment-MEM tool (v0.7.17-r1188) (Bolger et al., 2014; Li, 2013). Variant calling was carried out using the SAMtools/BCFtools (v1.10/v1.8) and the Genome Analysis Toolkit (v3.8–1-0-gf15c1c3ef) softwares using a minimum coverage depth cut-off of 20 (Li et al., 2009; McKenna et al., 2010). Only concordant variant calls from both methods were considered for downstream analysis. Variant classification was performed for mutations found across 27 drug resistance-associated genes (Supplementary Table 2) and annotated according to the clinical standard for molecular nomenclature (Phelan et al., 2019; den Dunnen et al., 2016). For resistance associated variants, and unless stated otherwise, all variants mentioned were detected at a minimum allelic frequency of 0.95. Sublineage assignment was carried out using TB-Profiler (v6.6.6) incorporating the latest SNP barcoding system proposed by Napier and colleagues (Napier et al., 2020). Genotypic DST (gDST) was also carried out with TBProfiler including standard prediction algorithm (Supplementary Table 3) and standard database found on the tbdb repository (https://github.com/jodyphelan/tbdb) (Phelan et al., 2019). *In silico* spoligotyping was performed with SpoTyping and clade assignment confirmed using SITVIT2 (Couvin et al., 2019; Xia et al., 2016).

### 2.3 Phylogenetics

A phylogenetic tree was constructed using 26,767 high-quality SNP sites (Coll et al., 2018). For quality assurance, SNP sites within low mappability loci (including proline-glutamic acid/proline-proline-glutamic acid protein-encoding genes and bacteriophage-derived genes) were removed. SNP sites in which the majority allele did not reach an allelic frequency of 0.75 were assigned as missing calls and sites with an excess of missing calls of 10% were removed. The R modelTest function (ape package) determined the Generalized Time-Reversible as the best nucleotide substitution model, validated under the Akaike information criterion (Schliep, 2011). The IQ-TREE software (v2.1.3) was used to construct a maximum-likelihood tree and the approximate likelihood-ratio test (aLRT) was used to provide branch support (Nguyen et al., 2015; Anisimova and Gascuel, 2006). The order of mutation acquisition was inferred in endemic clades from the tree topology and the distribution of mutations across the topological structure. Genomic clusters were identified using a 12 SNP cut-off from the genome-wide high-quality SNP set.

### 2.4 Minimum inhibitory concentration (MIC) determination

Twelve antimycobacterial drugs were tested (Table 1) utilizing a microdilution-based method, described as follows: 0.1 mL of Middlebrook 7H9 broth was first added to 96-well microplates, of which 8 wells were dedicated for testing a range of two-fold drug concentrations, while the remaining wells were used for growth controls at 100 and 1% mycobacterial density; negative controls and solvent controls for DMSO at 0.25% (maximum proportion tested), all in triplicate. Clinical isolates were incubated on LJ media slants for 3 to 4 weeks. Suspensions were prepared by first disaggregating a minimum of one loopful of mycobacteria into sterile tubes containing glass beads and vortexed vigorously. Following a 15-min resting period, the cells were resuspended in sterile water and adjusted to a 0.5 McFarland standard using a Grant Instruments Grant bio DEN-1B densitometer. Suspensions were diluted in Middlebrook 7H9 broth by a factor of 1:100 (100%) from which 0.1 mL were added to each well, reaching a final density of approximately 5 × 10^5^ CFU/mL. Additional suspensions with 1% of the density were also prepared by further diluting by a factor of 1:100, specifically for the 1% controls. Along with the clinical strains, MIC were also determined for the H37Rv reference strain (ATCC 27294) and the results expressed are concordant across three replicates done throughout the course of study at different timepoints. The inoculated microplates were incubated at 36 °C and visualized at days 7, 10 and 14, and MIC were recorded upon visible growth, equivalent to or greater than the growth of the 1% growth controls. Resistance phenotypes was evaluated taking into account the MIC and the respective epidemiological cut-off (ECOFF) or MGIT critical concentrations (CC). While the ECOFF represents the highest MIC within a wild-type (drug susceptible) population, the CC represents the lowest drug concentration necessary to inhibit at least 99% of the wild-type population (pyrazinamide: 90%) of a susceptible isolate. Since the European Committee on Antimicrobial Susceptibility Testing (EUCAST) is still gathering data and analyzing MIC distributions obtained by broth microdilution, only tentative (T) ECOFFs are available to some drugs (Supplementary Table 2).

**Table 1:** Isolates studied, lineage, spoligotypes (SIT/Clade), genomic cluster (GC, ≤12 SNP cutoff), relevant drug resistance-associated variants and MIC determined by broth microdilution for 12 anti-TB drugs.

Strain	Lineage	SIT/Clade	Cluster	Drug resistance-associated variants	Minimum inhibitory concentration (mg/L)											
					INH	RIF	RFB	EMB	STR	DCS	PAS	AMK	KAN	ETO	OFX	MXF
PT1	L4.3.4.2	SIT20/LAM1	GC1	*inhA* c.-15C > T, p.S94A; *iniA* p.P94fs; *rpoB* p.S450L; *rpsL* p. K43R	>4	>16	16	4	>32	16	1	1	5	>40	0.5	0.12
PT2	L4.1.1.3	SIT119/X1	NC	*rpoB* p.S450L; *gid* c.104del, p.L35fs	0.06	>16	16	2	4	16	≤0.5	1	2.5	1.25	0.25	0.12
PT3	L4.3.3	SIT64/LAM6	NC	ND	0.25	≤0.12	≤0.12	1	≤0.25	16	1	0.5	2.5	20	0.5	≤0.06
PT4	L4.3.4.2	SIT1106/LAM4	GC2	*inhA* c.-15C > T, p.I194T; *gid* p.A80P	2	≤0.12	≤0.12	1	2	16	1	1	2.5	>40	1	0.12
PT5	L4.3.4.2	SIT42/LAM9	NC	*iniA* p.P94fs; *katG* p.G494A	0.5	≤0.12	≤0.12	1	≤0.25	16	≤0.5	0.5	5	≤0.31	≤0.25	0.12
PT6	L4.3.4.2	SIT1106/LAM4	GC2	*embB* p.M306V; *inhA* c.-15C > T, p.I194T; *gid* p.A80P; *rpoB* p.S450L	4	>16	16	8	1	16	≤0.5	1	2.5	>40	≤0.25	0.12
PT7	L4.8	SIT2258	NC	*gyrB* p.D461H; *katG* p.S315T; *rpoB* p.S450L	4	>16	8	4	1	16	≤0.5	0.5	1.25	2.5	4	0.5
PT8	L4.3.3	SIT64/LAM6	NC	*inhA* c.-15C > T	0.5	≤0.12	≤0.12	2	0.5	16	≤0.5	0.5	1.25	>40	0.5	0.12
PT9	L4.3.4.2	SIT42/LAM9	NC	*embB* p.M306V; *katG* p.S315T; *rpoB* p.S450L; *rpsL* p.K43R	4	>16	8	8	>32	16	≤0.5	≤0.12	1.25	0.62	1	≤0.06
PT10	L4.3.4.2	SIT1106/LAM4	GC2	*embA* c.-16C > T; *embB* p.M306V; *inhA* c.-15C > T, p.I194T; *gid* p.A80P; *gyrA* p.D94A; *ribD* c.-12G > A; *rpoB* p.S450L; *rrs* n.1401A > G	4	>16	16	16	2	16	16	>16	>40	>40	4	1
PT11	L4.3.4.2	SIT20/LAM1	GC3	*inhA* c.-15C > T, p.S94A; *iniA* p.P94fs; *rpsL* p.K43R	2	≤0.12	≤0.12	1	>32	16	≤0.5	0.5	2.5	>40	≤0.25	≤0.06
PT12	L4.3.4.1	SIT20/LAM1	NC	*katG* p.S315T	4	≤0.12	≤0.12	2	2	16	2	0.5	2.5	0.62	0.5	0.25
PT13	L4.3.4.2	SIT2535	NC	*inhA* c.-15C > T	0.5	≤0.12	≤0.12	2	0.5	16	2	0.5	5	10	0.5	0.25
PT14	L2.2.1	SIT1/BEIJING	NC	*embB* p.D354A; *katG* p.S315T; *rpoB* p.S450L; *rpsL* p.K43R	2	>16	8	4	>32	32	≤0.5	0.5	1.25	20	≤0.25	≤0.06
PT15	L4.3.4.2	SIT1106/LAM4	GC2	*embB* p.M306V; *inhA* c.-15C > T, p.I194T; *gid* p.A80P; *rpoB* p.S450L	2	>16	>16	4	0.5	16	≤0.5	0.5	2.5	>40	1	0.12
PT16	L4.3.4.1	SIT20/LAM1	NC	*embA* c.-16C > A; *katG* p.S315T; *rpoB* p.S450L	4	>16	>16	4	≤0.25	16	4	0.5	2.5	1.25	0.5	0.12
PT17	L4.3.4.1	SIT20/LAM1	NC	*gid* p.W45*; *katG* p.S315T	4	≤0.12	≤0.12	2	2	16	1	0.5	2.5	1.25	0.5	0.12
PT18	L4.3.4.2	SIT20/LAM1	GC3	*inhA* c.-15C > T, p.S94A; *iniA* p.P94fs; *rpsL* p.K43R	2	≤0.12	≤0.12	1	>32	16	≤0.5	0.5	2.5	>40	0.5	0.12
PT19	L4.3.4.2	SIT81/LAM9	NC	*iniA* c.282_286del,p.P94fs	0.12	≤0.12	≤0.12	2	≤0.25	16	≤0.5	0.5	2.5	2.5	0.5	0.12
PT20	L2.2.1	SIT1/BEIJING	NC	*katG* p.S315T; *rpsL* p.K43R	>4	0.25	≤0.12	4	>32	32	2	1	2.5	2.5	0.5	0.25
PT21	L4.3.4.2	SIT20/LAM1	GC1	*alr* p.L113R; *eis* c.-10G > A; *embB* p.M306V; *gyrA* p.D94G; *inhA* c.-15C > T, p.S94A; *iniA* p.P94fs; *rpoB* p.S450L; *rpsL* p.K43R	4	>16	8	8	>32	32	≤0.5	2	20	>40	8	2
PT22	L4.3.4.1	SIT20/LAM1	NC	*inhA* c.-15C > T	0.5	≤0.12	≤0.12	1	0.5	16	≤0.5	1	5	5	≤0.25	0.12
PT23	L2.2.1	SIT1/BEIJING	NC	ND	≤0.03	0.12	≤0.12	1	≤0.25	4	≤0.5	0.5	1.25	0.62	0.25	0.06
PT24	L4.1.1.1	SIT137/X2	NC	ND	0.06	0.25	≤0.12	1	0.5	16	1	1	2.5	1.25	0.5	0.12
PT25	L4.3.4.1	SIT17/LAM2	NC	ND	0.12	0.25	≤0.12	2	0.5	16	1	1	5	1.25	0.5	0.25
PT26	L3	SIT357/CAS	NC	*ethA* p.G299_E311del; *katG* p.S315T; *rpoB* p.S450L; *rpsL* p.K43R	>4	>16	16	2	>32	16	2	1	2.5	5	0.5	≤0.06
PT27	L4.3.4.2	SIT20/LAM1	GC1	*alr* p.F4L; *eis* c.-10G > A; *embB* p.M306V; *gyrA* p.D94G; *inhA* c.-15C > T, p.S94A; *iniA* p.P94fs; *rpoB* p.S450L; *rpsL* p.K43R	>4	>16	8	8	>32	32	≤0.5	2	10	>40	8	4
PT28	L4.1.2.1	SIT53/T1	NC	*embB* p.M306V; *inhA* c.-15C > T; *katG* p.T380I; *rpoB* p.S450L	2	>16	>16	8	1	32	0.5	1	2.5	40	1	0.25
PT29	L2.2.1	SIT1/BEIJING	GC4	*embB* p.M306I; *katG* p.S315T; *rpoB* p.S450L; *rpsL* p.K43R	2	>16	8	4	>32	16	≤0.5	0.25	1.25	2.5	≤0.25	≤0.06
PT30	L4.3.4.2	SIT20/LAM1	NC	*inhA* c.-15C > T, p.S94A; *iniA* p.P94fs; *rpoB* p.D435V; *rpsL* p.K43R	4	>16	0.25	1	>32	16	≤0.5	0.5	2.5	>40	0.5	0.12
PT31	L2.2.1	SIT1/BEIJING	GC4	*embB* p.M306I; *katG* p.S315T; *rpoB* p.S450L; *rpsL* p.K43R	4	>16	8	2	>32	16	≤0.5	0.25	≤0.62	2.5	≤0.25	≤0.06
PT32	L4.2.1	SIT262/H3	NC	*embB* p.Q497K; *katG* p.S315T; *ribD* c.-12G > A; *rpoB* p.S450L; *rpsL* p.K88R; *rrs* n.1401A > G; *ubiA* p.V148A	>4	>16	>16	8	>32	16	16	>16	>40	5	1	0.25
PT33	L4.3.4.2	SIT1106/LAM4	GC2	*embB* p.M306V; *gid* p.A80P; *inhA* c.-15C > T, p.I194T; *rpoB* p.S450L	2	>16	16	8	2	16	1	0.5	2.5	>40	0.5	0.25
PT34	L4.3.4.2	SIT1106/LAM4	GC2	*embB* p.M306V; *gid* p.A80P; *inhA* c.-15C > T, p.I194T; *rpoB* p.S450L; *rrs* n.1401A > G	4	>16	16	4	0.5	8	≤0.5	>16	>40	>40	0.5	0.12
PT35	L4.3.4.2	SIT20/LAM1	GC3	*embB* p.Q497R; *inhA* c.-15C > T, p.S94A; *iniA* p.P94fs; *rpoB* p.S450L; *rpsL* p.K43R	>4	>16	8	8	>32	16	1	0.5	1.25	>40	≤0.25	0.12
PT36	L4.1	SIT53/T1	NC	*embB* p.M306I; *ethA* p.M1L; *gid* p.L79S; *inhA* c.-8 T > C; *katG* p.S315T; *rpoB* p.H445D	>4	>16	16	4	2	16	1	1	2.5	>40	0.5	0.12
PT37	L4.3.4.2	SIT20/LAM1	NC	*alr* p.M343T; *eis* c.-10G > A; *embA* c.-12C > A, c.-11C > A; *embB* p.P397T; *gyrA* p.S91P; *inhA* c.-15C > T, p.S94A; *iniA* p.P94fs; *rpoB* p.S450L; *rpsL* p.K43R; *rrs* n.1075_1076insT; *tlyA* p.L251fs	4	>16	>16	16	>32	32	≤0.5	>16	>40	>40	8	2
PT38	L4.3.4.2	SIT1106/LAM4	GC2	*embA* c.-16C > T; *embB* p.M306V; *gid* p.A80P; *gyrA* p.D94A; *inhA* c.-15C > T, p.I194T; *rpoB* p.S450L; *rrs* n.1401A > G	4	>16	>16	8	4	16	1	>16	>40	>40	4	2
PT39	L4.3.4.1	SIT20/LAM1	NC	ND	0.06	≤0.12	≤0.12	1	1	16	2	1	5	1.25	0.5	0.12
PT40	L4.1.1.1	SIT137/X2	NC	ND	0.06	≤0.12	≤0.12	2	≤0.25	16	1	0.5	2.5	2.5	0.5	0.12
H37Rv	—	—	NC	ND	0.06	≤0.12	≤0.12	2	≤0.25	16	≤0.5	0.25	≤0.62	1.25	0.5	0.12

INH, isoniazid; RIF, rifampin; RFB, rifabutin; EMB, ethambutol; STR, streptomycin; DCS, D-cycloserine; PAS, para-aminosalicylic acid; AMK, amikacin; KAN, kanamycin; ETO, ethionamide; OFX, ofloxacin; MXF, moxifloxacin; NC, not clustered; ND, not detected.

### 2.5 PK modeling and pharmacodynamic (PD) endpoints

Available population models describing each drug PK were searched in the literature and selected according to the population (adult patients not in intensive care unit), number of subjects, sampling scheme used and simplicity during development. The models and their respective identified co-variables for each drug are available in Supplementary Table 4. Following model replication using Berkeley Madonna, plasma profiles were simulated without variability for the standard TB patient (60 Kg male with normal renal function and dominant genotype), under each drug’s usual therapeutic regimen. This was accomplished through numerical integration of the models’ differential equations, with a time interval of 0.01 h until a steady stated was achieved, considering only the fixed parameters were adapted for the standard patient, based on the relevant co-variables (Nahid et al., 2016). Covariates that include renal and hepatic impairments, and potential drug interactions were not considered during simulations. Typically, the antimicrobial effect is dependent on both the concentration of drug in relation to the MIC and the time that this exposure is maintained. As such, several PK parameters, more or less directly related to the body’s exposure to the drug, were considered. The area under the curve (AUC), which indicates the total drug exposure over time, is frequently used when antibiotics have both concentration- and time-dependent effects, whereas the maximum concentration (Cmax) parameter, that only takes into account the peak plasma concentration of the drug, is used when the effect of concentration predominates over that of time. If, however, the antibiotic displays time-dependent effects, then the amount of time that the plasma concentration is above the MIC (t > MIC) or the lowest (trough) concentration observed in plasma just before the next drug administration (Cmin) are both frequently used. These PK parameters were either directly observed (Cmax, Cmin, t > MIC) or calculated by extending the integration of the steady state for 24 h (AUC) for the simulated profiles in each drug. An additional search in the literature was made for collecting clinical PD endpoints established for each drug, namely AUC/MIC, Cmax/MIC, Cmin/MIC or t > MIC, often considering also the free faction of the drug in plasma. Despite there being no consensus on optimal PD markers (the minimum value that the PD endpoint needs to show in order to conclude for efficacy) for the tested drugs, these were initially selected based on available clinical studies. If absent, PD markers were then selected from *in vitro* or *in vivo* pre-clinical studies. The basis, an EC_50_ (concentration of a drug that produces 50% of its maximal effect in the tested system) or EC_90_ (concentration of a drug required to achieve 90% of its maximal effect in the system) determined by an effective concentration sigmoid model, an observed bactericidal effect or an empirical approximation for the observed values, was also described. Lastly, PK parameters were combined with the MIC in order to evaluate qualitatively the efficacy of normal therapeutic regimens.

### 2.6 Statistical analysis

Differences in MIC distributions between drug-resistant strains belonging to specific genetic clades was assessed using the non-parametric Mann–Whitney test. Average MIC for each phylogenetic group and test statistics (*p*-values) are available in Supplementary Table 5. Statistical significance was considered for *p* < 0.05.

## 3 Results and discussion

### 3.1 Genetic diversity and drug resistance

This study entails the characterization of 40 clinical isolates, mostly drug-resistant strains (87.5%), of which 65.7% are MDR and 34.8% of these are pre-XDR (Table 1; Supplementary Table 1). Nearly all strains belong to the Latin-American-Mediterranean (LAM) clade, with the exception of 5 strains, belonging to the Beijing sublineage (lineage 2.2.1). Within the LAM subpopulation, 22.9% are classified as Lisboa3 (L4.3.4.2/LAM1/SIT20), while 20.0% are categorized as Q1 (L4.3.4.2/LAM4/SIT1106) (Table 1). A total of four genomic clusters were identified using a 12 SNP cut-off. Two clusters comprising a total of six isolates are associated with the Lisboa3 strains (GC1 and GC3) and highlight a higher genomic diversity within this clade, coupled with increased diversity of drug resistance associated variants, whereas the largest cluster (GC2), comprising seven isolates is associated with Q1 strains. A smaller Beijing cluster comprising two isolates was also detected. The genomic clusters found reflect the population structure of *M. tuberculosis* in Portugal associated with MDR and pre-XDR, usually dominated by Lisboa3 and Q1 strains albeit with increasing prevalence of Beijing strains (Perdigão et al., 2020; Perdigão et al., 2020).

### 3.2 Association between allelic configurations and drug resistance levels

The MICs to a total of 12 anti-TB drugs were determined for all isolates included in the study (*n* = 40). The MIC distribution obtained for each drug showed an overall good concordance with MGIT-based phenotypic DST (pDST) and gDST (Supplementary Figure 1), and are aligned with the global MIC distributions obtained using the EUCAST broth microdilution reference method (EUCAST, 2025). For some drugs the MIC distribution does not show a clear separation between wild-type susceptible isolates and drug-resistant isolates, for example: (i) for ETO, susceptible and resistant isolates showed overlapping MICs within the 2.5–5 mg/L range; or (ii) for DCS, although the observed MIC distribution fell within the range reported by EUCAST, the identification of a study-specific breakpoint was hampered since only one isolate was identified as DCS resistant by MGIT but had a MIC that corresponded to the mean MIC observed for susceptible wild-type isolates.

**Figure 1:**
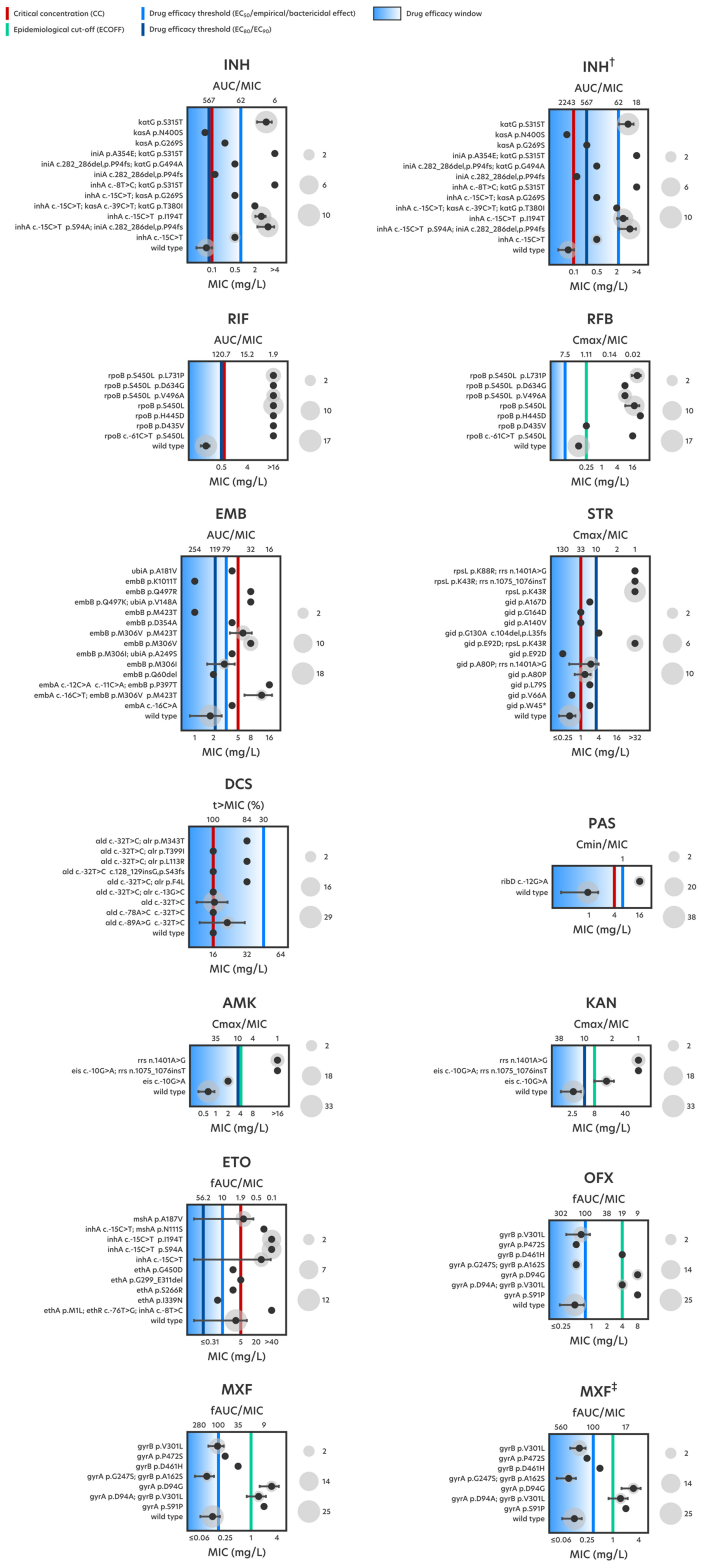
Drug resistance-associated genotypes, their respective average MIC and PD endpoints, for each tested drug. The MIC range predicted to remain under each drug’s effective killing potential is represented by a blue gradient. Gray circles denote the number of isolates harboring a given genotype. Results for high-doses of INH (†) and MXF (‡) are also shown.

**Figure 2:**
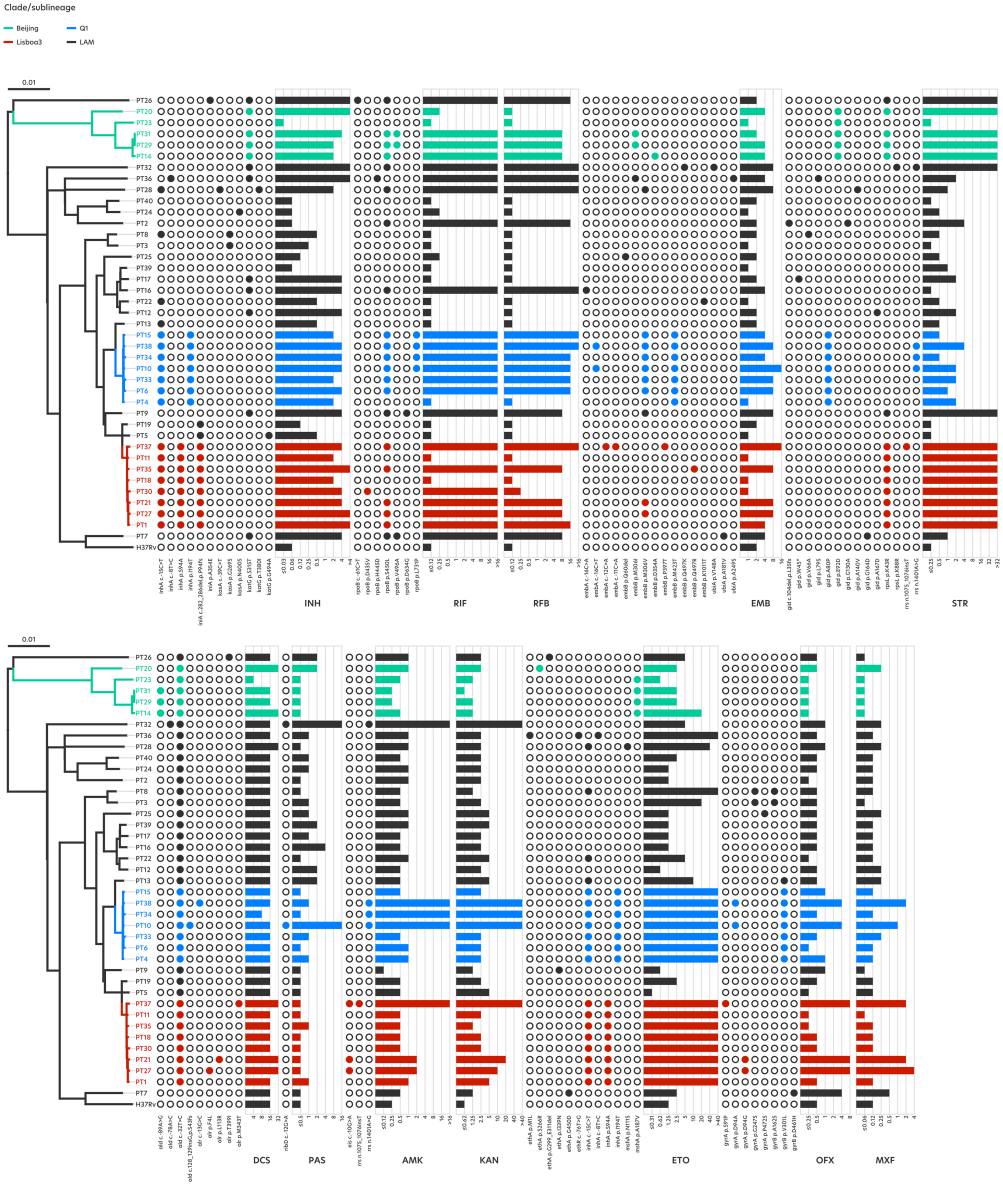
Phylogenetic tree of the 40 clinical strains from Portugal included in the study. Filled circles indicate presence of mutations. Bar plots indicate the strain’s respective MIC for each drug.

**Figure 3:**
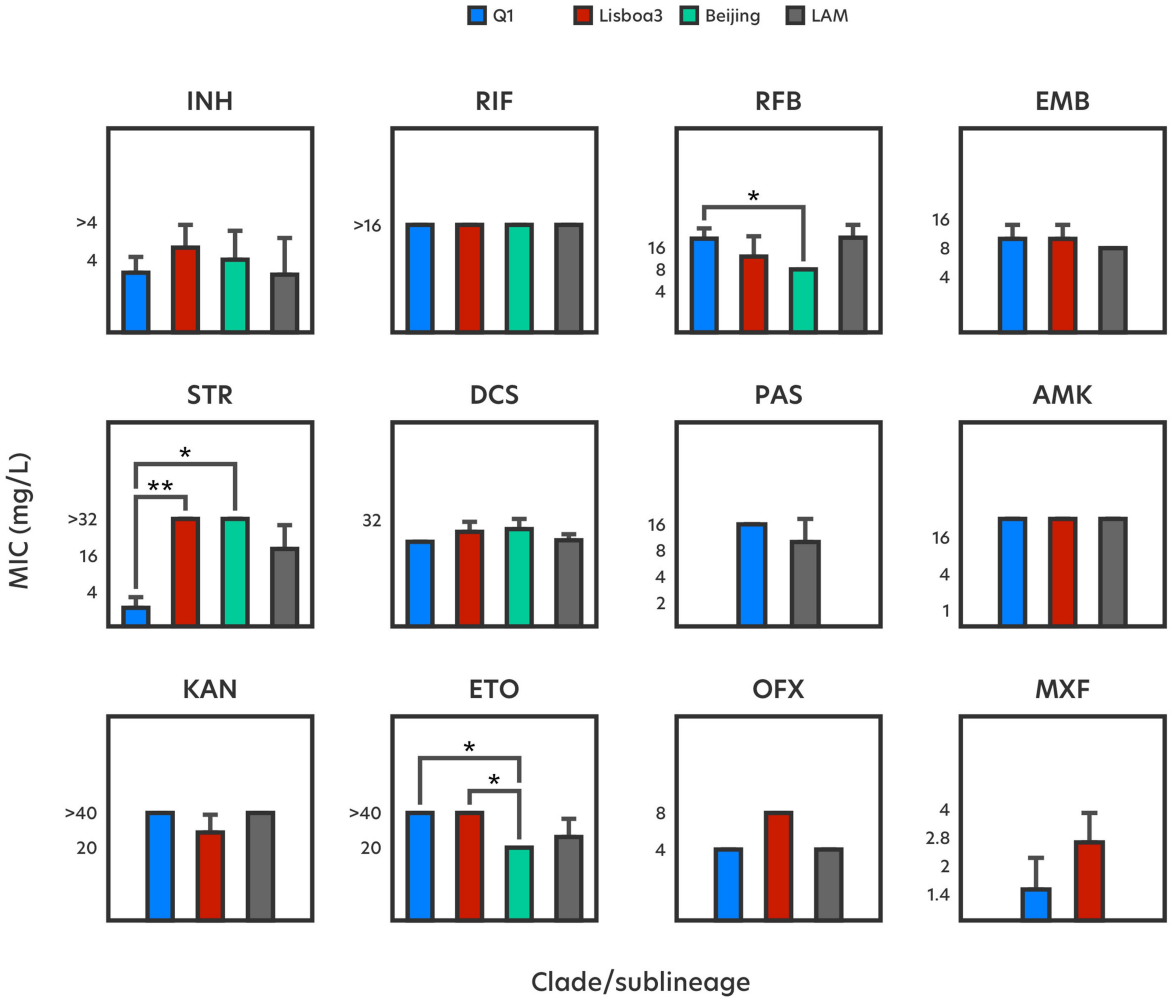
Average drug resistance levels by genetic clade. The comparison encompasses only isolates that show MICs above the ECOFF or, if unavailable, above the CC used in MGIT pDST. Statistically different pair-wise comparisons are highlighted by *p*-values (*) < 0.05 and (**) < 0.01.

With regards to INH, 12 (34%) drug-resistant strains that display high-level resistance (>2 mg/L) harbour mutations in the *katG* gene, particularly the S315T substitution (Figure 1). Mutations in the *inhA* gene are by far the most prevalent, occurring in either single or double mutation configurations across 19 (54%) INH-resistant strains, contributing to medium- to high-level INH resistance (0.5–>4 mg/L), respectively (Table 1). Double *inhA* mutations are characteristic among Q1 and Lisboa3 strains and herein found in 7 (20%) isolates displaying high-level resistance. According to the phylogenetic data, these strains appear to have evolved firstly by acquiring the *inhA* -15C > T promoter mutation followed by the acquisition of an *inhA* non-synonymous mutation (Figure 2). These particular microevolutionary molecular events have since constituted the main driver for the emergence of MDR-TB in Portugal, foregoing the need for *katG* missense mutations which are rarely present in the aforementioned endemic clades (Perdigão et al., 2014). These same combinations of *inhA* mutations also confer high-level resistance to the second-line functional analog ETO, although not to the same extent. While double *inhA* mutations contribute to a 6.3-fold increase in INH resistance compared to the average MIC attributed by single *inhA* mutation genotypes, the former only contributes to a much lower 2.5-fold increase in ETO resistance. The drug resistance-associated mutational background effects drug resistance levels differently among *M. tuberculosis* phylogenetic groups (Figure 3). Despite the differences in the levels of INH resistance between Q1, Lisboa3 and Beijing clades, these are not as pronounced and significant as the differences in the levels of ETO resistance, particularly between the endemic clades and Beijing strains. This is due to the lack of double *inhA* mutations within the Beijing subpopulation, which are the main drivers of high-level ETO resistance (>40 mg/L). In the case of INH, Beijing strains harbor high-level resistance-associated *katG* mutations instead, which are not associated with cross-resistance to ETO (Figure 2). One isolate (PT5) bore a G494A mutation in the *katG* gene whose association with INH resistance has been classified as uncertain while also showing a -48G > A promoter mutation in the *ahpC* gene which might be seen as a compensatory mutation (WHO, 2023).

Rifamycin-resistant strains harbor at least one mutation in the 81-bp rifampin resistance-determining region (RRDR)(Musser, 1995). The S450L mutation in the *rpoB* gene, highly frequent among rifamycin-resistant strains, and present in 21 (91%) RIF-resistant isolates appears as the main driver of high-level rifamycin resistance (≥8 mg/L) (Figure 1). Curiously, discrepancies in rifamycin resistance levels were found in an isolate harboring a single *rpoB* D435V mutation, contributing high- (>16 mg/L) and low-level (0.25 mg/L) resistance to RIF and rifabutin (RFB), respectively. Similar MICs have been reported for this mutation (Berrada et al., 2016). Significant differences in RFB resistance levels exist among Q1 strains and Beijing strains (Figure 3), seemingly unrelated with the drug resistance-associated mutational background of each group. Distinct combinations of double *rpoB* mutations exist in each set of strains (Figure 2), although, apart from those within the RRDR, the remaining *rpoB* mutations do not seem associated with rifamycin resistance (WHO, 2023), suggesting that other resistance-associated genes or intrinsic characteristics of these phylogenetic groups may be contributing to the differences in RFB resistance levels. One possibility pertains to the role and modulatory effect on drug resistance exerted by the efflux activity in these particular strains, which some of us have already demonstrated. In fact, we have previously demonstrated that efflux activity is more pronounced at MDR and pre-XDR strains, including strains belonging to the endemic clades herein studied, and does modulate the levels of resistance as part of a long-term effect response to drug exposure (Machado et al., 2018). However, further studies involving some of these mutations and evaluating the contribution of efflux in a larger sample across the microevolutionary trajectory of these isolates will be necessary to elucidate the mechanisms and additional molecular markers driving the observed differences in RIF resistance levels.

Still concerning RIF resistance, a total of 10 mutations other than those associated with RIF resistance were detected in *rpoB* and *rpoC* genes concomitantly with mutations in the RRDR, which could potentially act as compensatory mutations (Supplementary Table 6). From these, only two *rpoB* V496A and L731P mutations were found in more than one isolate: *rpoB* V496A was detected in three isolates, two from L2.2.1 and one from L4.8, highlighting its homoplasic nature; and, *rpoB* L731P, detected in four Q1 isolates after the acquisition of the S450L mutation (Supplementary Table 6). The latter was initially reported by Ioerger and colleagues in isolate X122, a pre-XDR strain from the Beijing R220 cluster in South Africa, thereby supporting its role as putative compensatory mechanism outside the helix bridge of the RNA polymerase’s *β*’ subunit (Ioerger et al., 2010; Meftahi et al., 2016).

The highest mutational diversity was observed for ethambutol (EMB) resistance-associated genes, with the most prevalent ones occurring within the codon 306 of the *embB* gene, present in 13 isolates, eight of which categorized as EMB-resistant by MGIT pDST. A previous characterization of *embB* mutations has also found the M306V and M306I mutations to be prevalent in clinical strains from Portugal, exclusive among the MDR subpopulation of EMB-resistant strains (Perdigão et al., 2009). Although these mutations were observed only in MDR strains, not all are associated with EMB resistance (Figure 2). The largest discrepancy between resistant phenotypes obtained through standard DST and the microdilution method occurs for EMB, where only 57.1% (8 in 14) of phenotypic EMB-resistant strains have MICs above the MGIT CC for EMB, which is the only possible comparison at this point considering there is still insufficient data to define an ECOFF for EMB by broth microdilution (Supplementary Table 2). High-level EMB resistance (≥8 mg/L) is associated with multiple-mutation genotypes involving at least one *embA* promoter mutation and at least one *embB* missense mutation (Figure 1). Given the ambiguity surrounding single and even double *embB* mutations, and their association with EMB resistance, *embA* promoter mutations, along with *embB* missense mutations appear to play a significant role in elevating EMB resistance levels. Careful consideration should be taken when interpreting genotypic DST results for EMB as single *embA* promoter mutations display MIC below the MGIT CC for EMB. This is especially relevant since this promoter mutation on its own may contribute to phenotypic resistance albeit undetected in standard DST (Cui et al., 2014). Our results corroborate this for the *embA* -16C > A promoter mutation in its single allelic configuration, with a MIC of 4 mg/L, insufficient for conferring EMB resistance (Figure 1). Between the two endemic clades, Q1 strains proportionally hold a greater number of *embB* missense mutations, whose microevolutionary path of acquisition is clear (Figure 2). The first *embB* mutation to be acquired was the M423T non-synonymous substitution, which does not appear to be associated with EMB resistance, followed by the acquisition of the *embB* M306V mutation, commonly associated with EMB resistance and shown to confer up to medium-level resistance (8 mg/L) (Figure 1).

High-level streptomycin (STR) resistance (>32 mg/L) is uniquely associated with *rpsL* mutations (Figure 1). Apart from these, *gid* mutations were found among 10 (25%) STR-resistant strains, solely associated with medium-level resistance (1–4 mg/L). The *gid* A80P mutation is Q1 clade-specific (Perdigão et al., 2014), and was found in both susceptible and resistant strains, conferring at best medium-level resistance (2–4 mg/L). Its acquisition predates the emergence of RIF resistance (Figure 2), indicating that Q1 strains were likely subjected to earlier regimens of anti-TB therapies. A significant difference in STR resistance levels is observed between Q1 resistant strains and resistant strains belonging to Lisboa3 and Beijing clades (Figure 3; Supplementary Table 4), due to the lack of high-level resistance-conferring *rpsL* mutations in the former.

A minority of 6 (15%) clinical isolates were found to exhibit cycloserine (DCS) MICs that appear to suggest resistance, albeit with low-level resistance (32 mg/L) (Figure 1) and yet none of the three for which routine MGIT-based pDST was done was classified as resistant. Among these, three resistance-associated mutations were detected in three isolates, the *alr* F4L, L113R and M343T substitutions (Figure 2). A previous genome-wide association study determined the *alr* gene to be significantly associated with DCS resistance, although the abovementioned mutations did not share this association, despite being high frequent alleles, suggesting that their selection is not dependent on drug resistance acquisition (Coll et al., 2018).

Two isolates (5%) showed high-level para-aminosalicylic acid (PAS) resistance (16 mg/L), both harboring the *ribD* -12G > A promoter mutation, although only one was resistant by MGIT pDST (Figure 1).

The second-line injectable agents AMK and KAN share identical resistance-associated genotypes. The predominant mutation in drug-resistant strains is the *rrs* 1401A > G mutation, found in 4 isolates, associated with high-level AMK (>16 mg/L) and KAN (>40 mg/L) resistance (Figure 1). A single *eis* -10G > A promoter mutation appears to contribute to medium-level AMK (2 mg/L) and KAN (10–20 mg/L) resistance, albeit differently when compared with a double-mutation configuration with a 1075_1076insT insertion in the *rrs* gene, leading to a 16.0-fold increase in AMK resistance, and only a 5.3-fold increase in KAN resistance. Similar to a previous study, which characterized the MIC of both aminoglycosides for a limited set of MDR strains from Portugal using a different methodology, isolates that harbored a single *eis* -10G > A promoter mutation showed MIC close to the CC and ECOFF for AMK (Perdigão et al., 2013). Aminoglycoside resistance appears to have been acquired differently between both endemic clades. While Q1 strains evolved by acquiring the *rrs* 1401A > G mutation, Lisboa3 strains happened to acquire the *eis* -10G > A promoter mutation, particularly effective at conferring high-level KAN resistance (Figure 2). Despite not having found any double *eis* mutations, studies show that the presence of mutations in both the promotor and coding regions of the *eis* gene may exhibit epistatic interactions that result in the loss of aminoglycoside resistance (Vargas et al., 2021). The diagnosis of these genotypes must be given special consideration as aminoglycosides could remain an effective therapeutic avenue, particularly against MDR TB.

Concerning fluoroquinolones, mutations were uncovered in the *gyrA* and *gyrB* genes. High-level ofloxacin (OFX) and moxifloxacin (MXF) resistance was observed mainly in genotypes consisting of the *gyrA* S91P (8 mg/L, 2 mg/L) and D94G (8 mg/L, 2–4 mg/L) mutations (Figure 1). Other mutations, such as *gyrA* D94A (4 mg/L, 1–2 mg/L) and *gyrB* D461H (4 mg/L, 0.5 mg/L) were also associated with medium- or high-level resistance. Considering that the proposed ECOFF for MXF elevates the CC for determining resistant phenotypes, the *gyrB* D461H mutation would no longer be associated with MXF resistance. Similarly, another mutation in codon 461 of the *gyrB* gene has been found associated with increased MIC for levofloxacin, an isomer of OFX, but not for MXF (The CRyPTIC Consortium, 2022). The *gyrA* D94A and S91P are specific mutational events that characterize the evolutionary process toward pre-XDR of the Q1 and Lisboa3 clades, respectively (Figure 2) (Perdigão et al., 2014).

The importance of correlating phenotyping with genotyping cannot be overstated and the association between mutations and drug resistance must be carefully reviewed before factoring into clinical decision-making. The WHO provides a catalogue of mutations and their association with phenotypic drug resistance, indispensable for diagnosing drug-resistant TB solely through genotypic methods (WHO, 2023). The in-depth analysis of the association of drug resistance levels with different allelic configurations presented in this study has established new relationships between mutations and drug resistance, whose addition in subsequent editions of said catalogue is highly recommended.

### 3.3 Drug resistance levels and its relationship with drug efficacy

The simulated PK models of each drug were utilized to ascertain the maximum concentration at which a drug remains efficacious.

At its recommended dose, INH is still effective against some drug resistance-associated genotypes. A high-dose of INH is proposed in cases of low-level resistance conferred by promoter or missense *inhA* mutations, in the absence of *katG* mutations (Daley and Chen, 2022). Although our data suggests an increased dose of INH is able to slightly overcome phenotypic resistance, it remains largely ineffective against these allelic configurations (Figure 1). Isoniazid is only half effective at treating INH-resistant strains harboring single *inhA* -15C > T promoter mutations. Therefore, choosing to treat these specific cases with a standard dose of INH does not suggest a successful treatment outcome.

Single *inhA* mutations appear to be sufficient for ensuring ETO resistance, given the predicted PK for this drug, suggesting its efficacy marker to fall significantly below the average MIC for genotypes consisting of single *inhA* -15C > T promoter mutations (Figure 1). Due to its overall low efficacy, even among strains harboring less prevalent *ethA* mutations, typically associated with low-level ETO resistance, its use should be reconsidered only against cases with phenotypical susceptibility to ETO devoid of mutations within ETO resistance-conferring genes.

As for EMB, the predicted PK model for this drug suggests an efficacy that ensures bacteriostatic effect for the majority of phenotypically EMB-susceptible strains harbouring mutations that are not associated with EMB resistance and those that are found in both phenotypes. This is the case for strains that have acquired a single M306I mutation in the *embB* gene, considering the mean MIC associated to this genotype is 3 mg/L, below the PD marker for EMB that indicates half effectiveness (Figure 1). Considering this, careful interpretation of phenotypic EMB resistance from *embB* mutations in codon 306 should be taken, especially given the prevalence of such mutations in both EMB-resistant and EMB-susceptible cases. Considering that the associated drug resistance levels tend to oscillate along its respective CC, single-allele configurations of *embB* M306 mutations are insufficient for confidently predicting EMB resistance.

Concerning STR, our data suggests that its standard dose is likely to remain efficacious against strains with medium-level resistance, conferred by single *gid* mutations (Figure 1). Reintegration of STR into anti-TB regimens could prove advantageous, particularly against clinically challenging extensively drug-resistant Q1 strains, which lack high-level STR resistance-associated mutations (Figure 2) (Perdigão et al., 2016).

Given DCS time-dependent killing activity, a 30% dosing interval is the minimum required for achieving bactericidal activity (Deshpande et al., 2018). It is predicted that this period encompasses MIC up to 45 mg/L, well above the tentative ECOFF of 32 mg/L for DCS (Figure 1). According to the recommended dose of 1,000 mg/d, it was shown that DCS can deliver a probability of target attainment (PTA) above 90% for a MIC of 16 mg/L (Chirehwa et al., 2020). Since the majority of clinical strains from Portugal (95%) demonstrated MIC around 16 mg/L, DCS may be considered an effective alternative or added treatment against TB.

Likewise, the group C drug PAS is predicted to be effective against almost every clinical isolate tested (Figure 1). The recommended dose for PAS of 12 g/d, administered once every 8 h, is predicted to achieve effectiveness under a minimum concentration of 6.3 mg/L. Similarly, a PK study has reported that the same dosage of 4 g every 8 h, lead to the highest minimum serological concentration of PAS whilst providing a PTA above 90% for MIC above 5 mg/L (de Kock et al., 2014). Consequently, PAS could be used as an alternative therapy against TB, since there is a high probability of clinical isolates from Portugal being susceptible to this drug.

According to our predicted PK models for both aminoglycosides, there are significant differences in drug efficacy between AMK and KAN extending beyond their respective proposed ECOFF. While AMK has a PD marker 3.49 times higher than its ECOFF, the one for KAN only extends 1.19 times beyond its ECOFF (Figure 1). This difference markedly influences each drug’s bactericidal potential, particularly with regards to AMK, which may still be effective against strains exhibiting low-to-medium resistance. This is evident as AMK appears to remain effective against strains harbouring the *eis* -10G > A promoter mutation, meanwhile KAN does not.

Some clinicians rely on higher doses of MXF to treat patients with fluoroquinolone-resistant TB and a MIC for MXF ranging between 1–2 mg/L (Daley and Chen, 2022). The majority of the isolates herein analyzed with high-level fluoroquinolone resistance would fall into this category, albeit our PK model predicts an increase in MXF dosage to provide only marginal benefits with regards to drug effectiveness. The standard MXF dosage of 0.4 g/d appears to be grossly insufficient for effective treatment of MDR TB, as some *gyrA/B* mutations, which are not typically associated with fluoroquinolone resistance may still increase tolerance to these drugs (Figure 1). Increasing the dosage may be beneficial for such cases and prevent the emergence of new drug resistance-associated mutations. Concomitantly, our PK model predicts that administrating a high-dose of MXF may ensure a peak plasma concentration well above the mutant prevention concentration of 8 mg/L and thus prevent the selection of MXF resistance-associated mutations, as it has already been shown in an *in vivo* study in which the administration of a higher dose of MXF can effectively prevent the emergence of MXF-resistant mutants (Almeida et al., 2007).

It is important to note that the PK/PD targets utilized in this study may not be strictly accurate given that there is evidence of variability within the same patient groups and the inter-strain, inter-laboratory and inter-methodology variability associated with the MIC measurements (Mouton et al., 2018). Regardless, this study provides a framework where the degree of uncertainty associated with technical variations in MIC determinations can be accommodated, allowing for a more detailed comparison and integration with selected PK/PD targets.

Considering that the clinical strains of *M. tuberculosis* analyzed in this study were isolated prior to the introduction of the latest anti-TB drugs bedaquiline, pretomanid and delamanid, these drugs were not included in the study. Nevertheless, an updated investigation should be pursued to determine the impact these drugs had on the genetic and epidemiological makeup of *M. tuberculosis* in Portugal, whether novel mutations were acquired as a consequence of the continued use of these drugs to treat MDR TB and pre-XDR TB and how effective these remain as drug resistance begins to emerge (Gómez-González et al., 2021).

## 4 Conclusion

The prevalence of the endemic Lisboa3 and Q1 strains, and their association with increased drug resistance levels present a serious obstacle in achieving effective treatment outcomes, given their overwhelming representation in MDR and pre-XDR TB cases currently circulating in Portugal. WGS enables simultaneous genome-wide characterization of drug resistance-associated mutations and in-depth epidemiological analysis of clinical isolates, providing invaluable information on which anti-TB therapeutic regimen is best suited. Genotyping is often a reliable strategy for rapid diagnosis drug resistance, although it is prone to false positives, highlighting the importance of utilizing phenotypic assays, particularly quantitative assays for drug resistance level characterization. Combined with clinical pharmacology, clinicians can select which drugs and what dosage should be administered for effective and personalized treatment of TB.

## Data Availability

The datasets presented in this study can be found in online repositories. The names of the repository/repositories and accession number(s) can be found in the article/Supplementary material.
