# Supplementary material for: The microevolutionary trajectory of endemic multidrug-resistant tuberculosis strains in Portugal toward increased drug resistance levels and its clinical significance
Source: Front Microbiol. 2026 Jan 21;16:1716549. doi: 10.3389/fmicb.2025.1716549 (PMC12868246; doi:10.3389/fmicb.2025.1716549)
Supplement: Supplementary file 1 [file Data_Sheet_1.PDF]

Supplementary Table 1 – Isolates studied, year of isolation, mapping statistics and categorical classification of phenotypic drug susceptibility testing by BACTEC MGIT. INH – Isoniazid, RIF – rifampin, PZA – pyrazinamide, EMB – ethambutol, STR – streptomycin, DCS – d-cycloserine, PAS – para-aminosalicylic acid, AMK – amikacin, KAN – kanamycin, CAP – capreomycin, ETO – ethionamide, OFX – ofloxacin, LZD – linezolid, CFZ – clofazimine. S – susceptible. R – resistant. NA – not available.

Strain	Year of isolation	Run accession	Mean read depth	Fold coverage	MGIT drug susceptibility testing													
					INH	RIF	PZA	EMB	STR	DCS	PAS	AMK	KAN	CAP	ETO	OFX	LZD	CFZ
PT1	2010	ERR14209629	125.22	99.38	R	R	S	S	R	S	S	S	NA	S	R	S	S	S
PT2	2010	ERR14209630	146.65	99.92	S	R	S	S	R	S	S	S	NA	S	S	S	S	S
PT3	2010	ERR14209631	49.68	99.50	R	S	S	S	S	NA	NA	NA	NA	NA	NA	NA	NA	NA
PT4	2010	ERR14209632	100.83	99.36	R	S	S	S	R	NA	NA	NA	NA	NA	NA	NA	NA	NA
PT5	2010	ERR14209633	79.37	99.26	R	S	S	S	S	NA	NA	NA	NA	NA	NA	NA	NA	NA
PT6	2010	ERR14209634	87.55	99.35	R	R	R	S	R	S	S	S	NA	S	R	S	S	S
PT7	2008	ERR14209635	63.83	99.84	R	R	R	S	S	S	S	S	NA	S	R	R	S	S
PT8	2008	ERR14209636	63.29	99.47	R	S	S	S	S	NA	NA	NA	NA	NA	NA	NA	NA	NA
PT9	2008	ERR14209637	81.95	99.33	R	R	R	S	R	S	S	S	NA	S	S	S	S	S
PT10	2008	ERR14209638	74.58	99.35	R	R	R	S	S	R	R	R	NA	R	R	R	S	S
PT11	2008	ERR14209639	78.83	99.43	R	S	S	S	R	NA	NA	NA	NA	NA	NA	NA	NA	NA
PT12	2008	ERR14209640	78.37	99.57	R	S	S	S	S	NA	NA	NA	NA	NA	NA	NA	NA	NA
PT13	2008	ERR14209641	166.62	99.36	R	S	S	S	S	NA	NA	NA	NA	NA	NA	NA	NA	NA
PT14	2008	ERR14209642	94.47	99.41	R	R	R	R	R	NA	NA	NA	NA	NA	NA	NA	NA	NA
PT15	2008	ERR14209643	86.76	99.36	R	R	R	S	S	NA	NA	NA	NA	NA	NA	NA	NA	NA
PT16	2009	ERR14209644	127.93	99.49	R	R	R	R	S	S	S	S	NA	S	S	R	S	S
PT17	2009	ERR14209645	126.82	99.38	R	S	S	S	R	NA	NA	NA	NA	NA	NA	NA	NA	NA
PT18	2009	ERR14209646	234.01	99.37	R	S	S	S	R	NA	NA	NA	NA	NA	NA	NA	NA	NA

PT19	2009	ERR14209647	59.25	99.35	R	S	S	S	S	NA	NA	NA	NA	NA	NA	NA	NA	NA
PT20	2009	ERR14209648	100.60	99.44	R	S	S	S	R	NA	NA	NA	NA	NA	NA	NA	NA	NA
PT21	2009	ERR14209649	156.83	99.36	R	R	R	R	R	S	S	S	NA	S	R	R	S	S
PT22	2009	ERR14209650	224.61	99.52	R	S	S	S	S	NA	NA	NA	NA	NA	NA	NA	NA	NA
PT23	2015	ERR14209651	525.58	99.64	S	S	S	S	S	NA	NA	NA	NA	NA	NA	NA	NA	NA
PT24	2015	ERR14209652	1138.49	99.83	S	S	S	S	S	NA	NA	NA	NA	NA	NA	NA	NA	NA
PT25	2015	ERR14209653	641.41	99.69	S	S	S	S	S	NA	NA	NA	NA	NA	NA	NA	NA	NA
PT26	2015	ERR14209654	1058.91	99.96	R	R	S	S	R	S	S	S	S	S	S	S	S	NA
PT27	2016	ERR14209655	690.25	99.65	R	R	R	R	R	S	S	S	R	S	R	R	S	NA
PT28	2010	ERR14209656	179.87	99.70	R	R	S	R	S	NA	NA	NA	NA	NA	NA	NA	NA	NA
PT29	2014	ERR14209657	1164.81	99.82	R	R	S	R	R	S	S	S	S	S	R	S	S	NA
PT30	2015	ERR14209658	439.02	99.49	R	R	R	R	R	S	S	S	S	S	R	S	S	NA
PT31	2014	ERR14209659	811.44	99.78	R	R	S	R	R	S	S	S	S	S	R	S	S	NA
PT32	2015	ERR14209660	412.10	99.86	R	R	R	R	R	S	S	R	R	R	S	S	S	NA
PT33	2015	ERR14209661	419.60	99.55	R	R	R	R	R	S	S	S	S	S	R	S	S	NA
PT34	2015	ERR14209662	387.04	99.48	R	R	R	R	R	S	S	R	R	R	R	S	S	NA
PT35	2015	ERR14209663	898.90	99.64	R	R	R	R	R	S	S	S	S	S	R	S	S	NA
PT36	2015	ERR14209664	592.15	99.83	R	R	S	S	R	S	S	S	S	S	R	S	S	NA
PT37	2009	ERR14209665	61.73	99.35	R	R	R	R	R	S	S	S	NA	R	R	R	S	NA
PT38	2008	ERR14209666	192.11	99.38	R	R	R	R	R	S	S	R	R	R	R	R	S	NA
PT39	2009	ERR14209667	256.90	99.42	S	S	S	S	S	NA	NA	NA	NA	NA	NA	NA	NA	NA
PT40	2009	ERR14209668	546.52	99.59	S	S	S	S	S	NA	NA	NA	NA	NA	NA	NA	NA	NA

Supplementary Table 2 – Critical concentrations (CC) used in MGIT, (tentative [T]) epidemiological cut-offs (ECOFF) for the drugs for which MIC were determined in the study and the genes screened for mutations. ID – insufficient data. † Provisional MGIT CC.

Drug	MGIT CC (mg/L)	Broth microdilution (T)ECOFF (mg/L)	Genes screened
INH	0.1 (1)	ID	*ahpC, inhA, iniA, kasA, katG, mshA*
RIF	0.5 (1)	ID	*rpoB, rpoC*
RFB	0.5 (2)	0.25 (3)	*rpoB, rpoC*
EMB	5 (4)	ID	*embA, embB, embC, embR, ubiA*
STR	1 (4)	ID	*gid, rpsL, rrs*
DCS	16 (5)	ID	*ald, alr*
PAS	4† (6)	ID	*folC, ribD, thyA, thyX*
AMK	1 (4)	4 (3)	*eis, rrs*
KAN	2.5 (7)	8 (3)	*eis, rrs*
ETO	5 (4)	ID	*ethA, ethR, inhA, mshA*
OFX	2 (7)	4 (3)	*gyrA, gyrB*
MXF	0.25 (4)	1 (3)	*gyrA, gyrB*

Supplementary Table 3 – Categorical classification of genotypic drug susceptibility testing through TBProfiler (v6.6.6) (8). MXF – moxifloxacin, BDQ – bedaquiline, PTM – pretomanid, DLM – delamanid.

Strain	Genotypic drug susceptibility testing																	
	INH	RIF	PZA	EMB	STR	DCS	PAS	AMK	KAN	CAP	ETO	OFX	MXF	LZD	CFZ	BDQ	PTM	DLM
PT1	R	R	S	S	R	S	S	S	S	S	R	S	S	S	S	S	S	S
PT2	S	R	S	S	R	S	S	S	S	S	S	S	S	S	S	S	S	S
PT3	R	S	S	S	S	S	S	S	S	S	R	S	S	S	S	S	S	S
PT4	R	S	S	R	R	S	S	S	S	S	R	S	S	S	S	S	S	S
PT5	R	S	S	S	S	S	S	S	S	S	S	S	S	S	S	S	S	S
PT6	R	R	R	R	R	S	S	S	S	S	R	S	S	S	S	S	S	S
PT7	R	R	R	S	S	S	S	S	S	S	S	R	R	S	S	S	S	S
PT8	R	S	S	S	S	S	S	S	S	S	R	S	S	S	S	S	S	S
PT9	R	R	R	R	R	S	S	S	S	S	S	S	S	S	S	S	S	S
PT10	R	R	R	R	R	R	S	R	R	R	R	R	R	S	S	S	S	S
PT11	R	S	S	S	R	S	S	S	S	S	R	S	S	S	S	S	S	S
PT12	R	S	S	S	S	S	S	S	S	S	S	S	S	S	S	S	S	S
PT13	R	S	S	S	S	S	S	S	S	S	R	S	S	S	S	S	S	S
PT14	R	R	R	R	R	S	S	S	S	S	R	S	S	S	S	S	R	R
PT15	R	R	R	R	R	S	S	S	S	S	R	S	S	S	S	S	S	S
PT16	R	R	S	S	S	S	S	S	S	S	S	S	S	S	S	S	S	S
PT17	R	S	S	S	R	S	S	S	S	S	S	S	S	S	S	S	S	S
PT18	R	S	S	S	R	S	S	S	S	S	R	S	S	S	S	S	S	S
PT19	S	S	S	S	S	S	S	S	S	S	S	S	S	S	S	S	S	S
PT20	R	S	S	S	R	S	S	S	S	S	S	S	S	S	S	S	S	S

PT21	R	R	R	R	R	R	S	S	R	S	R	R	R	S	S	S	S	S
PT22	R	S	S	S	S	S	S	S	S	S	R	S	S	S	S	S	S	S
PT23	S	S	S	S	S	S	S	S	S	S	S	S	S	S	S	S	S	S
PT24	S	S	S	S	S	S	S	S	S	S	S	S	S	S	S	S	S	S
PT25	S	S	S	S	S	S	S	S	S	S	S	S	S	S	S	S	S	S
PT26	R	R	S	S	R	S	S	S	S	S	S	S	S	S	S	S	S	S
PT27	R	R	R	R	R	R	S	S	R	S	R	R	R	S	S	S	S	S
PT28	R	R	S	R	S	S	S	S	S	S	R	S	S	S	S	S	S	S
PT29	R	R	S	R	R	S	S	S	S	S	R	S	S	S	S	S	S	S
PT30	R	R	R	S	R	S	S	S	S	S	R	S	S	S	S	S	S	S
PT31	R	R	S	R	R	S	S	S	S	S	R	S	S	S	S	S	S	S
PT32	R	R	S	R	R	S	S	R	R	R	R	S	S	S	S	S	S	S
PT33	R	R	R	R	R	S	S	S	S	S	R	S	S	S	S	S	S	S
PT34	R	R	R	R	R	S	S	R	R	R	R	S	S	S	S	S	S	S
PT35	R	R	R	R	R	S	S	S	S	S	R	S	S	S	S	S	S	S
PT36	R	R	R	R	R	S	S	S	S	S	R	S	S	S	S	S	S	S
PT37	R	R	R	R	R	R	S	S	R	R	R	R	R	S	R	R	S	S
PT38	R	R	R	R	R	S	S	R	R	R	R	R	R	S	S	S	S	S
PT39	S	S	S	S	S	S	S	S	S	S	S	S	S	S	S	S	S	S
PT40	S	S	S	S	S	S	S	S	S	S	S	S	S	S	S	S	S	S

Supplementary Table 4 – Parameters used in pharmacokinetic (PK) modeling. Pharmacodynamic (PD) markers were selected based on different studies, whenever clinical studies were unavailable: (a) in vitro, (b) in vivo pre-clinical or (c) in vivo. † INH high-dose. ‡ MXF high-dose.

Drug	Dose (mg/day)	PK model		PK parameter	PD endpoint	PD marker	MIC determination model
		Model structure	Co-variables				
INH[a]	300	Ka, tlag, 2 comp. (9)	Weight on CL and Vd (9)	AUC (9): 46.7 mg·h/L	AUC/MIC (10)	62× (EC_{50}), 567× (EC_{90}) (10, 11)	Agar proportion method (12)
	900†			AUC (9): 140.2 mg·h/L			
RIF[a]	600	Transit, 1 comp., auto-induction (13)	Weight on CL and Vd (13)	AUC (13): 60.79 mg·h/L	AUC/MIC (14)	120.7× (EC_{90}) (14)	Agar proportion method (12)
RFB[c]	300	Ka, tlag, 2 comp. (15)	Weight on CL, V1, V2 and Q. Genotype for F (15)	Cmax (15): 0.278 mg/L	Cmax/MIC (16)	7.5× (empirical) (16)	Broth dilution method (17)
EMB[a]	1600	Ka, 1 comp. (18)	Weight on CL and Vd (18)	AUC (18): 21.2 mg·h/L	AUC/MIC (19)	79× (EC_{50}), 119× (EC_{90}) (19)	Agar proportion method (12)
STR[c]	15	Ka, 1 comp. (20)	Weight on CL and Vd (20)	Cmax (20): 32.6 mg/L	Cmax/MIC (21)	10× (EC_{90}) (21)	Bactec 460-TB radiometric method (22)
DCS[a]	500	Ka, tlag, 1 comp. (23)	Weight on Vd (23)	Cmax (23): N.A.	t>MIC (24)	30% (bactericidal effect) (11, 24)	Sensititre MYCOTB method (24)
PAS[c]	12000	Ka, 1 comp. (25)	–	Cmin (25): 6.3 mg/L	Cmin/MIC (26)	1× (empirical) (26)	Agar proportion method (12)
AMK[a]	15	1 comp. (27)	Weight on CL and Vd (27)	Cmax (27): 34.9 mg/L	Cmax/MIC (28)	10× (EC_{90}) (11, 28)	Broth dilution method (12)
KAN[c]	15	Ka, 1 comp. (25)	–	Cmax (25): 47.4 mg/L	Cmax/MIC (29)	10× (EC_{90}) (29)	Agar proportion method (12)
ETO[a]	500	Ka, tlag, 1 comp. (30)	Weight on CL and Vd (30)	fAUC (30): 9.7 mg·h/L	fAUC/MIC (31)	10× (EC_{50}), 56.2× (EC_{80}) (11, 31)	Sensititre MYCOTB method (31)

OFX[b]	800	Ka, tlag, 1 comp. (32)	Weight on CL and Vd (32)	fAUC (32): 75.5 mg·h/L	fAUC/MIC (33)	100× (bactericidal effect) (33)	Agar dilution method (33)
MXF[b]	400	Ka, tlag, 1 comp. Weight on CL and Vd (25)	Weight on CL (25)	fAUC (25): 17.49 mg·h/L	fAUC/MIC (34)	100× (bactericidal effect) (34)	Broth microdilution method (12)
	800[‡]			fAUC (25): 34.98 mg·h/L			

Supplementary Table 5 – Mann-Whitney test statistics and accompanied average MIC of each tested drug for every genetic clade. Statistically significant p-values are highlighted in bold.

Drug	Test statistic					MIC (mg/L)
	**Clade**	**Q1**	**Lisboa3**	**Beijing**	**LAM**	
INH	Q1	N.A.	0.189	0.917	0.579	3.14
	Lisboa3	0.189	N.A.	0.53	0.108	5
	Beijing	0.917	0.53	N.A.	0.468	4
	LAM	0.579	0.108	0.468	N.A.	3.03
RIF	Q1	N.A.	N.A.	N.A.	N.A.	32
	Lisboa3	N.A.	N.A.	N.A.	N.A.	32
	Beijing	N.A.	N.A.	N.A.	N.A.	32
	LAM	N.A.	N.A.	N.A.	N.A.	32
RFB	Q1	N.A.	0.108	**0.029**	0.061	18.3
	Lisboa3	0.108	N.A.	0.532	0.396	9.06
	Beijing	**0.029**	0.532	N.A.	0.97	4.85
	LAM	0.061	0.396	0.97	N.A.	8.88
EMB	Q1	N.A.	0.762	0.112	0.476	8
	Lisboa3	0.762	N.A.	0.072	0.26	8.8
	Beijing	0.112	0.072	N.A.	0.209	4
	LAM	0.476	0.26	0.209	N.A.	6
STR	Q1	N.A.	**0.001**	**0.013**	0.7	2.2
	Lisboa3	**0.001**	N.A.	N.A.	**0.005**	64
	Beijing	**0.013**	N.A.	N.A.	**0.039**	64
	LAM	0.7	**0.005**	**0.039**	N.A.	20.5
DCS	Q1	N.A.	N.A.	N.A.	N.A.	N.A.
	Lisboa3	N.A.	N.A.	N.A.	N.A.	32
	Beijing	N.A.	N.A.	N.A.	N.A.	32
	LAM	N.A.	N.A.	N.A.	N.A.	32
PAS	Q1	N.A.	N.A.	1	0.265	16
	Lisboa3	N.A.	N.A.	N.A.	N.A.	N.A.
	Beijing	1	N.A.	N.A.	0.755	2
	LAM	0.265	N.A.	0.755	N.A.	4.67
AMK	Q1	N.A.	0.793	0.505	0.074	19.6
	Lisboa3	0.793	N.A.	0.456	0.058	9.25
	Beijing	0.505	0.456	N.A.	1	1
	LAM	0.074	0.058	1	N.A.	4.44
KAN	Q1	N.A.	0.119	N.A.	**0.037**	80

	Lisboa3	0.119	N.A.	N.A.	0.185	28.75
	Beijing	N.A.	N.A.	N.A.	N.A.	N.A.
	LAM	**0.037**	0.185	N.A.	N.A.	17.5
ETO	Q1	N.A.	N.A.	**0.023**	**0.007**	80
	Lisboa3	N.A.	N.A.	**0.013**	**0.004**	80
	Beijing	**0.023**	**0.013**	N.A.	1	20
	LAM	**0.007**	**0.004**	1	N.A.	30.62
OFX	Q1	N.A.	0.096	N.A.	N.A.	4
	Lisboa3	0.096	N.A.	N.A.	0.248	8
	Beijing	N.A.	N.A.	N.A.	N.A.	N.A.
	LAM	N.A.	0.248	N.A.	N.A.	4
MXF	Q1	N.A.	0.333	N.A.	N.A.	1.5
	Lisboa3	0.333	N.A.	N.A.	N.A.	2.67
	Beijing	N.A.	N.A.	N.A.	N.A.	N.A.
	LAM	N.A.	N.A.	N.A.	N.A.	N.A.

Supplementary Table 6 – Most relevant drug resistance-associated variants and putative compensatory mutations for strains with other mutations as the underlaying cause of drug resistance. ND – not detected.

Strain	Drug resistance-associated variants	Putative compensatory mutations			
		ahpC	***rpoA***	***rpoB***	***rpoC***
PT1	inhA c.-15C>T, p.S94A; iniA p.P94fs; rpoB p.S450L; rpsL p.K43R	ND	ND	ND	ND
PT2	*rpoB* p.S450L; *gid* c.104del,p.L35fs	ND	ND	ND	p.G594E, p.D747G
PT3	ND	ND	ND	ND	ND
PT4	*inhA* c.-15C>T, p.I194T; *gid* p.A80P	ND	ND	ND	ND
PT5	*iniA* p.P94fs; *katG* p.G494A	c.-48G>A	ND	ND	ND
PT6	*embB* p.M306V; *inhA* c.-15C>T, p.I194T; *gid* p.A80P; *rpoB* p.S450L	ND	ND	ND	ND
PT7	*gyrB* p.D461H; *katG* p.S315T; *rpoB* p.S450L	ND	ND	p.V496A	ND
PT8	*inhA* c.-15C>T	ND	ND	ND	ND
PT9	*embB* p.M306V; *katG* p.S315T; *rpoB* p.S450L; *rpsL* p.K43R	ND	ND	p.D634G	p.G442C
PT10	embA c.-16C>T; embB p.M306V; inhA c.-15C>T, p.I194T; gid p.A80P; gyrA p.D94A; ribD c.-12G>A; rpoB p.S450L; rrs n.1401A>G	ND	ND	p.L731P	ND
PT11	*inhA* c.-15C>T, p.S94A; *iniA* p.P94fs; *rpsL* p.K43R	ND	ND	ND	ND
PT12	*katG* p.S315T	ND	ND	ND	ND
PT13	*inhA* c.-15C>T	ND	ND	ND	ND
PT14	*embB* p.D354A; *katG* p.S315T; *rpoB* p.S450L; *rpsL* p.K43R	ND	ND	ND	ND
PT15	*embB* p.M306V; *inhA* c.-15C>T, p.I194T; *gid* p.A80P; *rpoB* p.S450L	ND	ND	p.L731P	ND
PT16	*embA* c.-16C>A; *katG* p.S315T; *rpoB* p.S450L	ND	ND	ND	ND
PT17	*gid* p.W45*; *katG* p.S315T	ND	ND	ND	ND
PT18	*inhA* c.-15C>T, p.S94A; *iniA* p.P94fs; *rpsL* p.K43R	ND	ND	ND	ND
PT19	*iniA* c.282_286del,p.P94fs	ND	ND	ND	ND

PT20	*katG* p.S315T; *rpsL* p.K43R	ND	ND	ND	ND
PT21	*alr* p.L113R; *eis* c.-10G>A; *embB* p.M306V; *gyrA* p.D94G; *inhA* c.-15C>T, p.S94A; *iniA* p.P94fs; *rpoB* p.S450L; *rpsL* p.K43R	ND	ND	ND	ND
PT22	*inhA* c.-15C>T	ND	ND	ND	ND
PT23	ND	ND	ND	ND	p.E1092D
PT24	ND	ND	ND	ND	p.G594E
PT25	ND	p.P44R	ND	ND	ND
PT26	*ethA* p.G299_E311del; *katG* p.S315T; *rpoB* p.S450L; *rpsL* p.K43R	c.-88G>A	ND	c.-61C>T	ND
PT27	*alr* p.F4L; *eis* c.-10G>A; *embB* p.M306V; *gyrA* p.D94G; *inhA* c.-15C>T, p.S94A; *iniA* p.P94fs; *rpoB* p.S450L; *rpsL* p.K43R	ND	ND	ND	ND
PT28	*embB* p.M306V; *inhA* c.-15C>T; *katG* p.T380I; *rpoB* p.S450L	ND	ND	ND	p.G594E
PT29	*embB* p.M306I; *katG* p.S315T; *rpoB* p.S450L; *rpsL* p.K43R	ND	ND	p.V496A	ND
PT30	*inhA* c.-15C>T, p.S94A; *iniA* p.P94fs; *rpoB* p.D435V; *rpsL* p.K43R	ND	ND	ND	ND
PT31	*embB* p.M306I; *katG* p.S315T; *rpoB* p.S450L; *rpsL* p.K43R	ND	ND	p.V496A	ND
PT32	*embB* p.Q497K; *katG* p.S315T; *ribD* c.-12G>A; *rpoB* p.S450L; *rpsL* p.K88R; *rrs* n.1401A>G; *ubiA* p.V148A	ND	ND	ND	p.P1040R
PT33	*embB* p.M306V; *gid* p.A80P; *inhA* c.-15C>T, p.I194T; *rpoB* p.S450L	ND	ND	ND	p.V483G
PT34	*embB* p.M306V; *gid* p.A80P; *inhA* c.-15C>T, p.I194T; *rpoB* p.S450L; *rrs* n.1401A>G	ND	ND	p.L731P	ND
PT35	*embB* p.Q497R; *inhA* c.-15C>T, p.S94A; *iniA* p.P94fs; *rpoB* p.S450L; *rpsL* p.K43R	ND	ND	ND	ND
PT36	*embB* p.M306I; *ethA* p.M1L; *gid* p.L79S; *inhA* c.-8T>C; *katG* p.S315T; *rpoB* p.H445D	ND	ND	ND	p.G594E
PT37	*alr* p.M343T; *eis* c.-10G>A; *embA* c.-12C>A, c.-11C>A; *embB* p.P397T; *gyrA* p.S91P; *inhA* c.-15C>T, p.S94A; *iniA* p.P94fs; *rpoB* p.S450L; *rpsL* p.K43R; *rrs* n.1075_1076insT; *tlyA* p.L251fs	ND	ND	ND	p.E49Q, p.K1152Q
PT38	*embA* c.-16C>T; *embB* p.M306V; *gid* p.A80P; *gyrA* p.D94A; *inhA* c.-15C>T, p.I194T; *rpoB* p.S450L; *rrs* n.1401A>G	ND	ND	p.L731P	ND
PT39	ND	ND	ND	ND	ND
PT40	ND	ND	ND	ND	p.G594E
H37Rv	ND	ND	ND	ND	ND

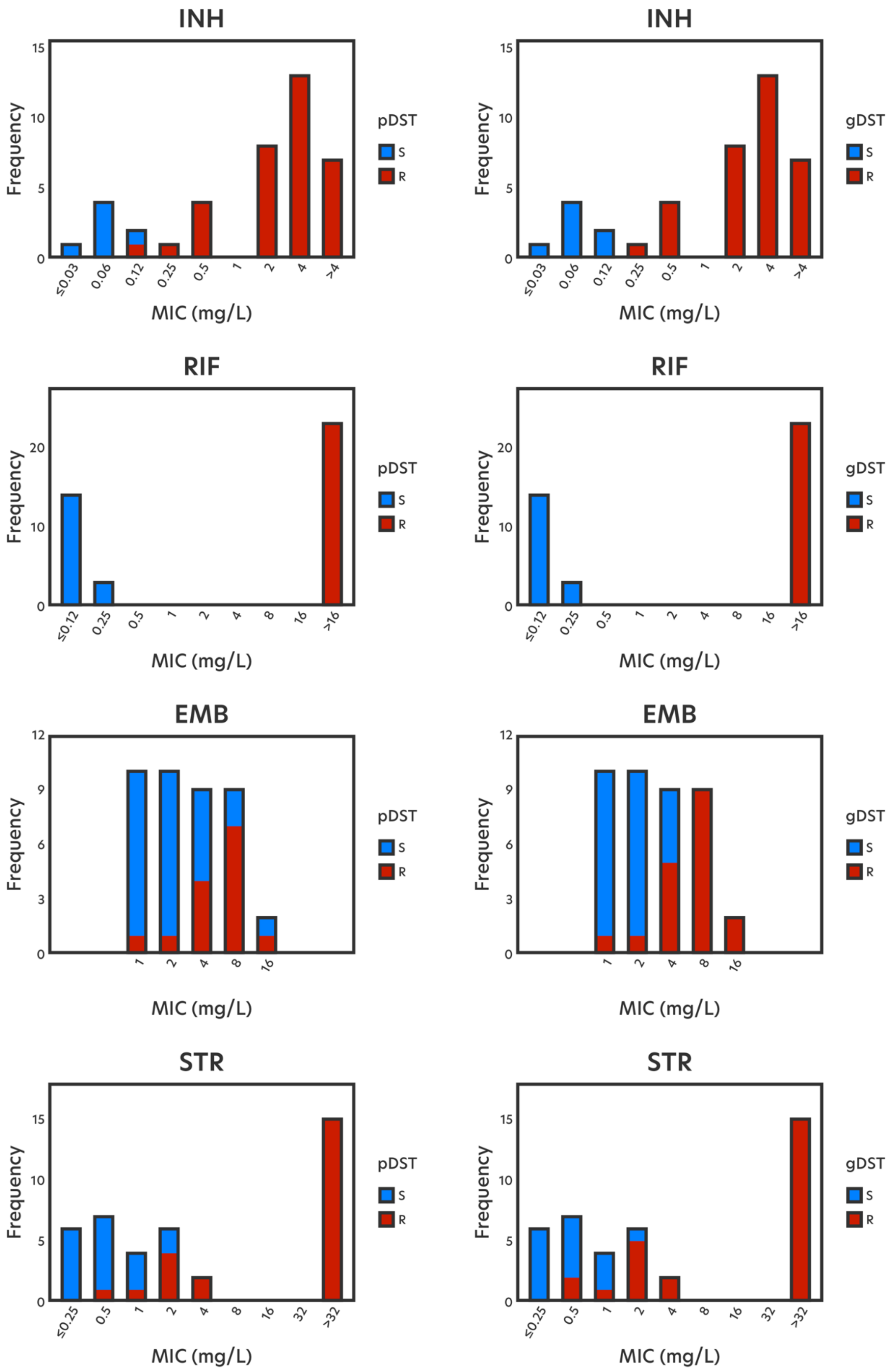

Supplementary Figure 1 – MIC distributions of susceptible (S) and resistant (R) strains by (left) phenotypic DST (pDST) (BACTEC MGIT) and (right) genotypic DST (gDST) (TBProfiler). ND – not determined.

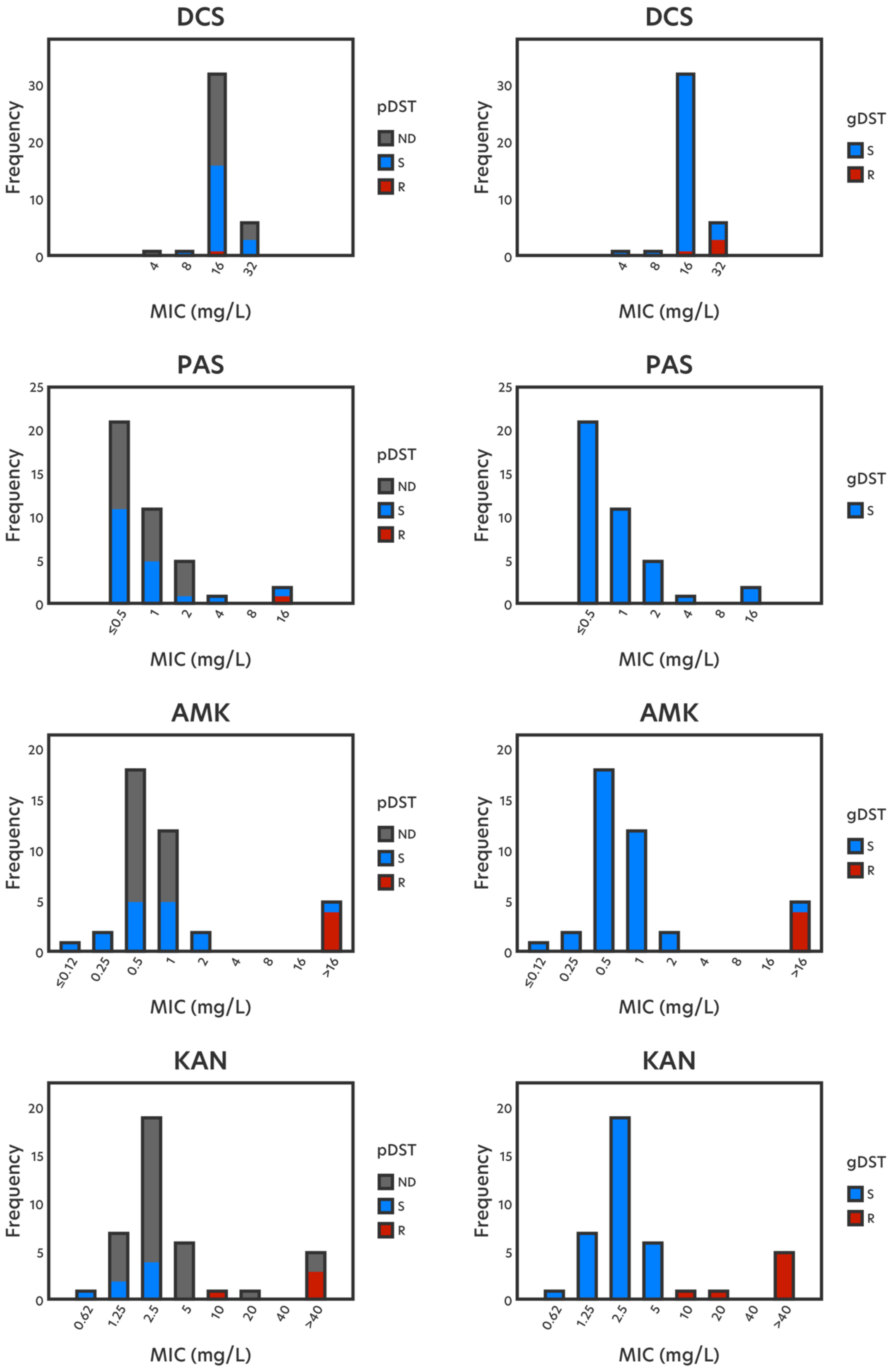

Supplementary Figure 1 (Cont.)

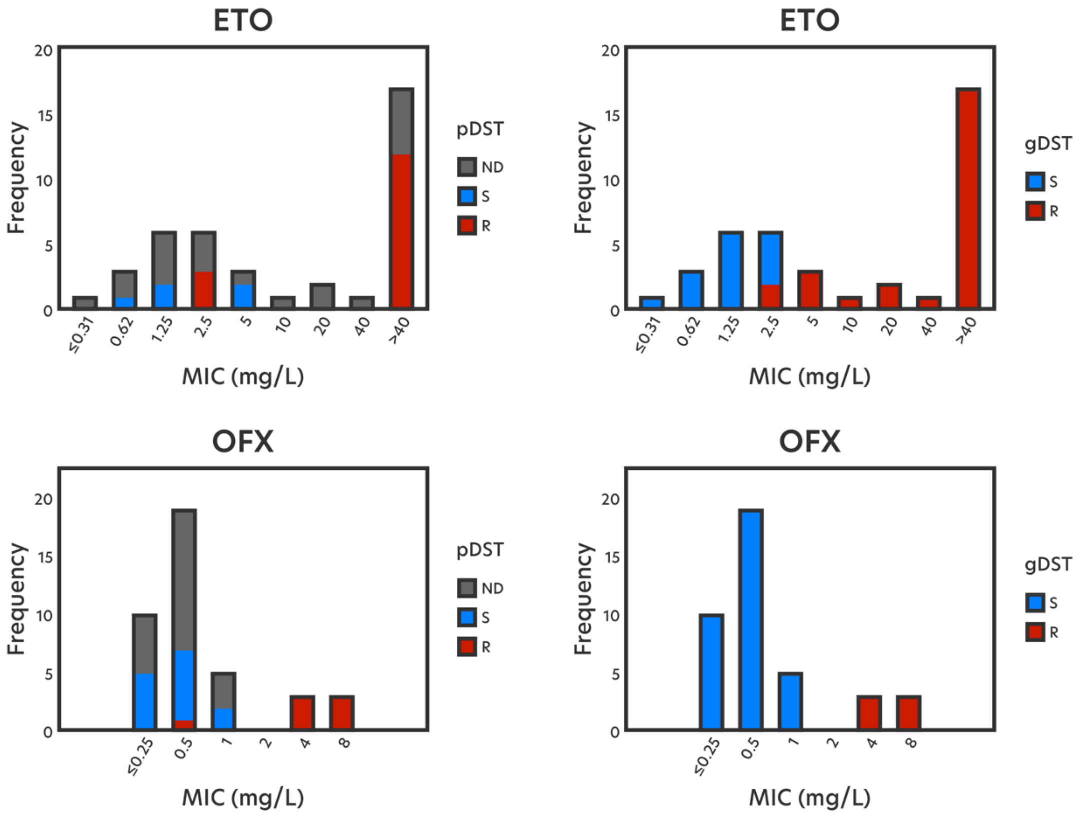

Supplementary Figure 1 (Cont.)

REFERENCES

1. World Health Organization (WHO). Technical report on critical concentrations for drug susceptibility testing of isoniazid and the rifamycins (rifampicin, rifabutin and rifapentine). Geneva; 2021.
2. Rüsch-Gerdes S, Pfyffer GE, Casal M, Chadwick M, Siddiqi S. Multicenter laboratory validation of the BACTEC MGIT 960 technique for testing susceptibilities of Mycobacterium tuberculosis to classical second-line drugs and newer antimicrobials. J Clin Microbiol. 2006;44(3):688-92.
3. European Committee on Antimicrobial Susceptibility Testing (EUCAST). Antimicrobial wild type distributions of microorganisms 2025 [Available from: https://www.mic.eucast.org/search/.
4. World Health Organization (WHO). Technical manual for drug susceptibility testing of medicines used in the treatment of tuberculosis. Geneva; 2018.
5. World Health Organization (WHO). WHO operational handbook on tuberculosis. Module 3: diagnosis - rapid diagnostics for tuberculosis detection, third edition. Web Annex B. Critical concentrations for pretomanid and cycloserine: WHO policy statement. Geneva; 2024.
6. Mansjö M, Espinosa-Gongora C, Samanci I, Groenheit R, Werngren J. Performance of a broth microdilution assay for routine minimum inhibitory concentration determination of 14 anti-tuberculous drugs against the Mycobacterium tuberculosis complex based on the EUCAST reference protocol. Antimicrob Agents Chemother. 2025;69(2):e0094624.
7. World Health Organization (WHO). Technical Report on critical concentrations for drug susceptibility testing of medicines used in the treatment of drug-resistant tuberculosis. Geneva; 2018.
8. Phelan JE, O'Sullivan DM, Machado D, Ramos J, Oppong YEA, Campino S, et al. Integrating informatics tools and portable sequencing technology for rapid detection of resistance to anti-tuberculous drugs. Genome Med. 2019;11(1):41.
9. Rodriguez CA, Zuluaga AF, Neely MN, Sierra Y, Morales-Gutierrez J, Zapata J, et al. Nonparametric Population Pharmacokinetic Modeling of Isoniazid in Colombian Patients With Tuberculosis. Ther Drug Monit. 2019;41(6):719-25.
10. Gumbo T, Louie A, Liu W, Brown D, Ambrose PG, Bhavnani SM, et al. Isoniazid bactericidal activity and resistance emergence: integrating pharmacodynamics and pharmacogenomics to predict efficacy in different ethnic populations. Antimicrob Agents Chemother. 2007;51(7):2329-36.

11. Alffenaar JC, de Steenwinkel JEM, Diacon AH, Simonsson USH, Srivastava S, Wicha SG. Pharmacokinetics and pharmacodynamics of anti-tuberculosis drugs: An evaluation of in vitro, in vivo methodologies and human studies. Front Pharmacol. 2022;13:1063453.
12. Woods GL, Brown-Elliott BA, Desmond EP, Hall GS, Heifets LB, Pfyffer GE, et al. Susceptibility Testing of Mycobacteria, Nocardiae, and Other Aerobic Actinomycetes; Approved Standard. NCCLS document M24-A. 940 West Valley Road, Suite 1400, Wayne, Pennsylvania 19087-1898 USA: NCCLS; 2003.
13. Jing Y, Zhu LQ, Yang JW, Huang SP, Wang Q, Zhang J. Population Pharmacokinetics of Rifampicin in Chinese Patients With Pulmonary Tuberculosis. J Clin Pharmacol. 2016;56(5):622-7.
14. Gumbo T, Louie A, Deziel MR, Liu W, Parsons LM, Salfinger M, et al. Concentration-dependent Mycobacterium tuberculosis killing and prevention of resistance by rifampin. Antimicrob Agents Chemother. 2007;51(11):3781-8.
15. Hennig S, Naiker S, Reddy T, Egan D, Kellerman T, Wiesner L, et al. Effect of SLCO1B1 Polymorphisms on Rifabutin Pharmacokinetics in African HIV-Infected Patients with Tuberculosis. Antimicrob Agents Chemother. 2016;60(1):617-20.
16. Davies G, Cerri S, Richeldi L. Rifabutin for treating pulmonary tuberculosis. Cochrane Database Syst Rev. 2007;2007(4):Cd005159.
17. Heifets LB, Lindholm-Levy PJ, Iseman MD. Rifabutine: minimal inhibitory and bactericidal concentrations for Mycobacterium tuberculosis. Am Rev Respir Dis. 1988;137(3):719-21.
18. Zhu M, Burman WJ, Starke JR, Stambaugh JJ, Steiner P, Bulpitt AE, et al. Pharmacokinetics of ethambutol in children and adults with tuberculosis. Int J Tuberc Lung Dis. 2004;8(11):1360-7.
19. Srivastava S, Musuka S, Sherman C, Meek C, Leff R, Gumbo T. Efflux-pump-derived multiple drug resistance to ethambutol monotherapy in Mycobacterium tuberculosis and the pharmacokinetics and pharmacodynamics of ethambutol. J Infect Dis. 2010;201(8):1225-31.
20. Zhu M, Burman WJ, Jaresko GS, Berning SE, Jelliffe RW, Peloquin CA. Population pharmacokinetics of intravenous and intramuscular streptomycin in patients with tuberculosis. Pharmacotherapy. 2001;21(9):1037-45.
21. Streptomycin. Tuberculosis (Edinb). 2008;88(2):162-3.
22. Rastogi N, Labrousse V, Goh KS. In vitro activities of fourteen antimicrobial agents against drug susceptible and resistant clinical isolates of Mycobacterium tuberculosis and comparative intracellular activities against the virulent H37Rv strain in human macrophages. Curr Microbiol. 1996;33(3):167-75.

23. Alghamdi WA, Alsultan A, Al-Shaer MH, An G, Ahmed S, Alkabab Y, et al. Cycloserine Population Pharmacokinetics and Pharmacodynamics in Patients with Tuberculosis. Antimicrob Agents Chemother. 2019;63(5).

24. Deshpande D, Alffenaar JC, Köser CU, Dheda K, Chapagain ML, Simbar N, et al. d-Cycloserine Pharmacokinetics/Pharmacodynamics, Susceptibility, and Dosing Implications in Multidrug-resistant Tuberculosis: A Faustian Deal. Clin Infect Dis. 2018;67(suppl_3):S308-s16.

25. Chang MJ, Jin B, Chae JW, Yun HY, Kim ES, Lee YJ, et al. Population pharmacokinetics of moxifloxacin, cycloserine, p-aminosalicylic acid and kanamycin for the treatment of multi-drug-resistant tuberculosis. Int J Antimicrob Agents. 2017;49(6):677-87.

26. de Kock L, Sy SK, Rosenkranz B, Diacon AH, Prescott K, Hernandez KR, et al. Pharmacokinetics of para-aminosalicylic acid in HIV-uninfected and HIV-coinfected tuberculosis patients receiving antiretroviral therapy, managed for multidrug-resistant and extensively drug-resistant tuberculosis. Antimicrob Agents Chemother. 2014;58(10):6242-50.

27. Dijkstra JA, van Altena R, Akkerman OW, de Lange WC, Proost JH, van der Werf TS, et al. Limited sampling strategies for therapeutic drug monitoring of amikacin and kanamycin in patients with multidrug-resistant tuberculosis. Int J Antimicrob Agents. 2015;46(3):332-7.

28. Srivastava S, Modongo C, Siyambalapitiyage Dona CW, Pasipanodya JG, Deshpande D, Gumbo T. Amikacin Optimal Exposure Targets in the Hollow-Fiber System Model of Tuberculosis. Antimicrob Agents Chemother. 2016;60(10):5922-7.

29. Strydom N, Gupta SV, Fox WS, Via LE, Bang H, Lee M, et al. Tuberculosis drugs' distribution and emergence of resistance in patient's lung lesions: A mechanistic model and tool for regimen and dose optimization. PLoS Med. 2019;16(4):e1002773.

30. Al-Shaer MH, Märtson AG, Alghamdi WA, Alsultan A, An G, Ahmed S, et al. Ethionamide Population Pharmacokinetic Model and Target Attainment in Multidrug-Resistant Tuberculosis. Antimicrob Agents Chemother. 2020;64(9).

31. Deshpande D, Pasipanodya JG, Mpagama SG, Srivastava S, Bendet P, Koeuth T, et al. Ethionamide Pharmacokinetics/Pharmacodynamics-derived Dose, the Role of MICs in Clinical Outcome, and the Resistance Arrow of Time in Multidrug-resistant Tuberculosis. Clin Infect Dis. 2018;67(suppl_3):S317-s26.

32. Stambaugh JJ, Berning SE, Bulpitt AE, Hollender ES, Narita M, Ashkin D, et al. Ofloxacin population pharmacokinetics in patients with tuberculosis. Int J Tuberc Lung Dis. 2002;6(6):503-9.

33. Chigutsa E, Meredith S, Wiesner L, Padayatchi N, Harding J, Moodley P, et al. Population pharmacokinetics and pharmacodynamics of ofloxacin in South African patients with multidrug-resistant tuberculosis. Antimicrob Agents Chemother. 2012;56(7):3857-63.

34. Shandil RK, Jayaram R, Kaur P, Gaonkar S, Suresh BL, Mahesh BN, et al. Moxifloxacin, ofloxacin, sparfloxacin, and ciprofloxacin against Mycobacterium tuberculosis: evaluation of in

vitro and pharmacodynamic indices that best predict in vivo efficacy. Antimicrob Agents Chemother. 2007;51(2):576-82.
